# Composition, function, and timing: exploring the early-life gut microbiota in piglets for probiotic interventions

**DOI:** 10.1186/s40104-023-00943-z

**Published:** 2023-11-13

**Authors:** Jianping Quan, Cineng Xu, Donglin Ruan, Yong Ye, Yibin Qiu, Jie Wu, Shenping Zhou, Menghao Luan, Xiang Zhao, Yue Chen, Danyang Lin, Ying Sun, Jifei Yang, Enqin Zheng, Gengyuan Cai, Zhenfang Wu, Jie Yang

**Affiliations:** 1 College of Animal Science and National Engineering Research Center for Breeding Swine Industry, South China Agricultural University, Guangzhou, Guangdong People’s Republic of China; 2 Guangdong Provincial Key Laboratory of Agro-Animal Genomics and Molecular Breeding, South China Agricultural University, Guangzhou, Guangdong China; 3 Yunfu Subcenter of Guangdong Laboratory for Lingnan Modern Agriculture, Yunfu, Guangdong China; 4 National Engineering Research Center for Breeding Swine Industry, Wens Foodstuff Group Co., Ltd., Yunfu, Guangdong People’s Republic of China

**Keywords:** Colonization resistance, Intestinal microbiota, Piglet, Weaning, Weight gain

## Abstract

**Background:**

The establishment of a robust gut microbiota in piglets during their early developmental stage holds the potential for long-term advantageous effects. However, the optimal timeframe for introducing probiotics to achieve this outcome remains uncertain.

**Results:**

In the context of this investigation, we conducted a longitudinal assessment of the fecal microbiota of 63 piglets at three distinct pre-weaning time points. Simultaneously, we gathered vaginal and fecal samples from 23 sows. Employing 16S rRNA gene and metagenomic sequencing methodologies, we conducted a comprehensive analysis of the fluctuation patterns in microbial composition, functional capacity, interaction networks, and colonization resistance within the gut microbiota of piglets. As the piglets progressed in age, discernible modifications in intestinal microbial diversity, composition, and function were observed. A source-tracking analysis unveiled the pivotal role of fecal and vaginal microbiota derived from sows in populating the gut microbiota of neonatal piglets. By D21, the microbial interaction network displayed a more concise and efficient configuration, accompanied by enhanced colonization resistance relative to the other two time points. Moreover, we identified three strains of *Ruminococcus* sp. at D10 as potential candidates for improving piglets' weight gain during the weaning phase.

**Conclusions:**

The findings of this study propose that D10 represents the most opportune juncture for the introduction of external probiotic interventions during the early stages of piglet development. This investigation augments our comprehension of the microbiota dynamics in early-life of piglets and offers valuable insights for guiding forthcoming probiotic interventions.

**Supplementary Information:**

The online version contains supplementary material available at 10.1186/s40104-023-00943-z.

## Background

The gut microbiota plays an important role in modulating the health and physiological functions of both humans and animals [1, 2]. In mammals, the colonization of the gut microbiota is conventionally believed to commence subsequent to the birth of a neonate, albeit differing viewpoints exist [3, 4]. Postpartum, progeny gradually acquire microorganisms from various sources, including the maternal vaginal tract, skin, and external environment [5, 6]. These microbial inhabitants within the gut exhibit continual adaptability to the evolving immune and physiological state of the host during distinct growth stages [7]. Within this progression, the composition and function of the microbial community undergo substantial modifications in correspondence with the host's characteristics [8, 9]. Over time, this dynamic interplay leads to the formation of a gut microbial community that establishes a reciprocal symbiosis with the host and demonstrates resilience against external perturbations.

Nevertheless, despite the considerable fluctuations in the gut microbiota during diverse stages of mammalian life, certain bacteria are more prone to exert a persistent influence on host health and growth after their colonization during early-life. For example, Martínez et al. [10] demonstrated that the sequence of colonization influences the establishment of specific bacterial groups and their enduring presence, particularly evident in mice with less diverse initial microbial communities. Another study indicated that the favorable microbiome assembled in early life can impact the immune equilibrium and overall well-being of mice into adulthood [11]. This phenomenon, termed "priority effects", underscores how the order and timing of arrival of new species within a community can shape their establishment [12]. During the process of microbiome assembly, organisms arriving early often undergo niche preemption, particularly through competitive utilization of nutrients, a phenomenon known as exploitative competition. This process plays a crucial role in shaping the composition and dynamics of the microbiota [13, 14]. To summarize, microorganisms that establish themselves in the host’s early-life are more likely to secure suitable niche opportunities compared to external colonizers when the gut microbiota has reached a state of dynamic equilibrium. Consequently, bacteria arrving later may encounter heightened colonization resistance [15, 16]. Thus, seizing the "opportunity window" of intestinal microbiome assembly in the initial phases of offspring existence to artificially facilitate the estabilishment of beneficial gut microbiota holds paramount significance, particularly in agricultural animals such as pig.

In the context of pig husbandry, the supplementation of beneficial bacteria to feed is a common practice aimed at enhancing growth performance and mitigating piglet diarrhea through improved nutritional digestion and intestinal barrier function [17]. Findings from studies by Martine et al. [10] and Fulde et al. [11] indicate that incorporating beneficial bacteria in the early stages of life is more advantageous. The early life of a pig typically encompasses its lactation period, encompassing significant milestones such as birth, introduction of creep feed, and weaning, in modern pig farming. However, limited research exists to ascertain the optimal time point during this early phase for the introduction of external probiotics. Therefore, the objectives of this study are as follows: 1) To scrutinize the composition and function of the gut microbiota during three pivotal time points in the early life of pigs. 2) To delve into the interactions and colonization resistance of the gut microbiota at distinct time points during lactation, building upon the insights derived from the first objective. 3) To pinpoint the most opportune timing for the introduction of beneficial microorganisms, with the intention of optimizing the pig's gut microbial community and fostering enduring health and growth performance. The outcomes of this study are poised to provide significant insights into the developmental trajectory of the gut microbiota in pigs.

## Methods

### Study design and sample collection

**Fig. 1:**
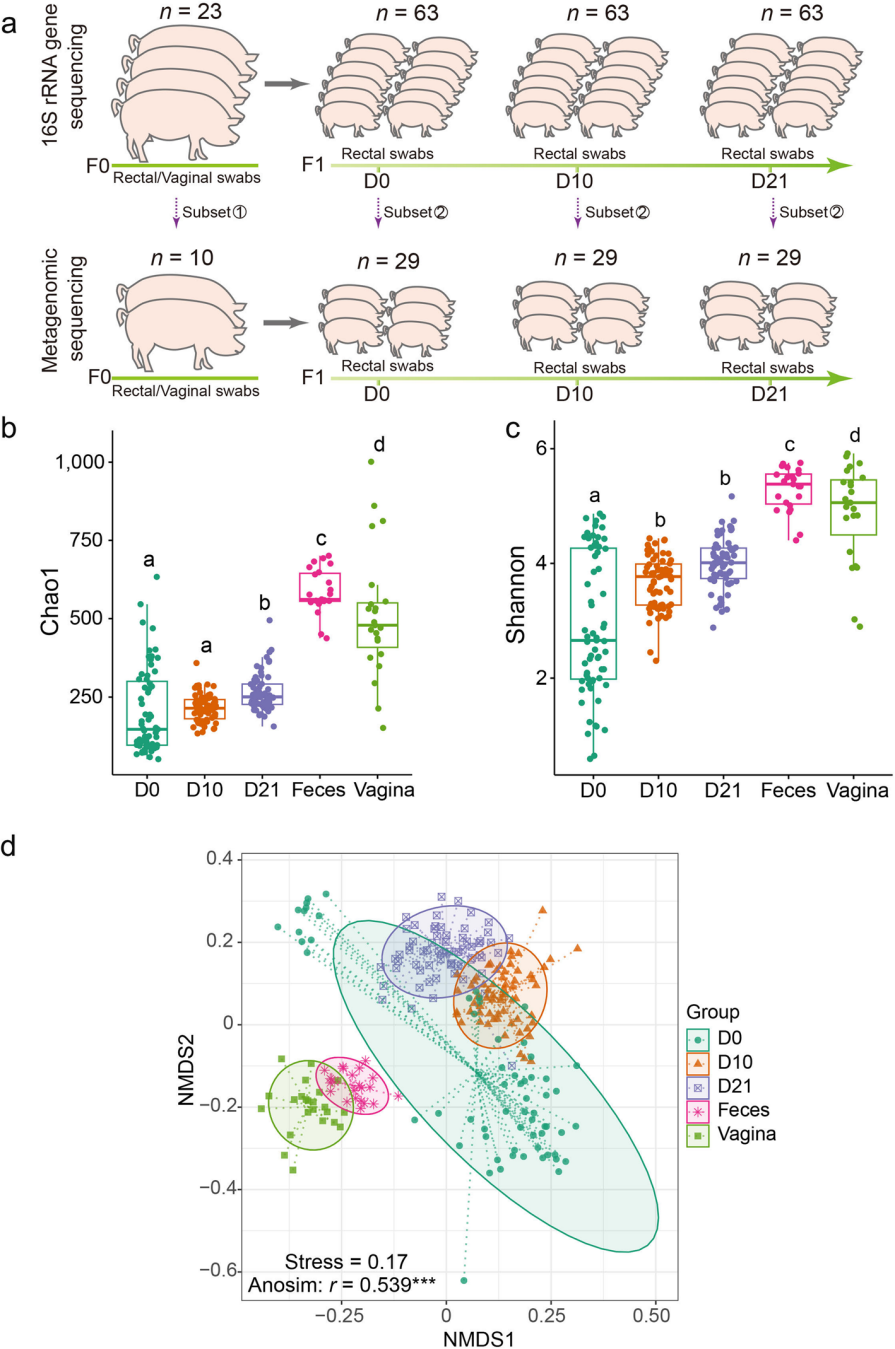
Overview of the experimental design and diversity of microbial community among different groups. **a** Overall workflow of sample collection from piglets, and sows at each sampling time point. All sows and their piglets were co-raised in the same environment. All swab samples were used for 16S rRNA gene sequencing, and a subset of samples was randomly selected for metagenomic sequencing. **b** The Chao1 index. **c** The Shannon index. Each point represents one sample as indicated by the axis label and color of the point. Letters (a, b, c, d) above the boxplots indicate significant differences between the two groups (*P* < 0.05). **d** Non-metric Multidimensional Scaling (NMDS) plot according sample group based on the abundance of ASVs. The plot is based on the Bray–Curtis distances between pairs of samples. Each point represents one sample; ellipses represent the 95% confidence for all points within each cluster. Stress, the value used to estimate the NMDS ordination fitness. R, the statistic from the analysis of similarities (ANOSIM) that compares the mean of ranked dissimilarities between groups to the mean of ranked dissimilarities within groups. *P*, the *P*-value from ANOSIM analysis between groups

A total of 23 Licha sows and their progeny (135 piglets) were included in this research endeavor. The Licha black pig, an indigenous Chinese breed, serves as the parental lineage, while the piglets are the outcome of crossbreeding between Yorkshire boars and Licha sows. In this study, the pigs were housed with sows in neighboring farrowing rooms, and both the sows and their offspring coexisted within the same area. The flooring within the farrowing areas was constructed with cast iron slatted flooring. At the time of parturition, rectal and vaginal swab samples were collected from all 23 sows. Similarly, rectal swab samples from the piglets (totaling 135) were procured at three distinct intervals during lactation: upon colostrum intake (referred to as D0), one day post-creep feed introduction (D10), and the day of weaning (D21). These swabs were promptly preserved at −80 °C until the subsequent DNA extraction process. D0 samples were collected within 5 h of piglet birth. During this timeframe, the piglets and sows were co-located, and the piglets had unrestricted access to maternal milk. It is noteworthy that the creep feed diet employed throughout the study was devoid of antibiotics, and the birth and weaning weight of each piglet were recorded at D0 and D21, respectively. Furthermore, the piglets stemming from each sow were collectively housed within a shared enclosure (as illustrated in Fig. 1a). A cumulative total of 323 rectal swab samples were obtained from the 135 piglets across the aforementioned three time points. However, swab samples from only 63 piglets encompassed all three time points, as some piglets were omitted from the sample pool due to encountering episodes of diarrhea during their growth trajectory. Consequently, a total of 189 microbial samples (63 samples from each of the time points: D0, D10, and D21) were subjected to subsequent 16S rRNA gene sequencing.

In tandem with the microbial investigation, an additional facet of the research involved metagenomic analysis targeting a subset of the cohort. More specifically, a subset of 10 sows was randomly selected from the initial group of 23, and within this context, a total of 29 piglets (each contributing rectal swab samples across all three time points) born to these 10 sows were enlisted for the metagenomic analysis (as depicted in Fig. 1a). This phase culminated in a cumulative sum of 235 samples earmarked for 16S rRNA gene sequencing and 107 samples allocated for metagenomic sequencing.

### DNA extraction, PCR amplification, and 16S rRNA gene sequencing

Extraction of microorganism DNA from the 235 rectal swab samples was undertaken using the CTAB/sodium dodecyl sulfate (SDS) method, a modified iteration of the CTAB (hexadecyltrimethylammonium bromide) technique [18]. Notably, the CTAB/SDS method entails the introduction of SDS during the lysis phase [19], augmenting cell wall disintegration and expediting efficient protein removal. The purity and concentration of the extracted DNA were assessed by NanoDrop (Thermo Scientific, Massachusetts, USA) and agarose gel electrophoresis (Novogene, Tianjin, China). DNA samples adhering to the ensuing criteria successfully cleared the quality control assessment: a 260/280 ratio ranging from 1.8 to 2.0, a concentration equal to or exceeding 10 ng/μL, and a principal band fragment size on the gel of no less than 500 bp. Following successful quality control, the DNA samples were suitably diluted with sterile water, reaching a concentration of 1 ng/μL. The V3-V4 segment of the bacterial 16S rRNA gene was subjected to amplification using universal primers 341F: 5'-CCTAYGGGRBGCASCAG-3' and 806R: 5'-GGACTACNNGGGTATCTAAT-3' [20]. To construct the libraries, the barcode sequence specific to each sample was synthesized along with the universal primer sequence to form the final primer sequence used for library amplification [21]. Each PCR consisted of 15 µL of Phusion® High-Fidelity PCR Master Mix (New England Biolabs, Ipswich, Massachusetts, USA), 0.2 µmol/L of each primer, and 10 ng of target DNA. The thermal cycling regimen encompassed an initial denaturation at 98 °C for 1 min, followed by 30 cycles of denaturation at 98 °C for 10 s, annealing at 50 °C for 30 s, and elongation at 72 °C for 30 s. The protocol concluded with a final elongation phase of 5 min at 72 °C. The size of the amplicons was verified through 2% agarose gel electrophoresis (Novogene, Tianjin, China). PCR products were mixed in equidensity ratios. Then, mixture PCR products were purified with Qiagen Gel Extraction Kit (Qiagen, Hilden, Germany). The purified amplicons were further used for library preparation and ensuing sequencing via the TruSeq DNA PCR-Free Sample Preparation Kit (Illumina, San Diego, California, USA) in accordance with the manufacturer's protocol. The quality of the library was assessed via Qubit@ 2.0 Fluorometer (Thermo Scientific, Waltham, Massachusetts, USA) and an Agilent Bioanalyzer 2100 system (Agilent, Santa Clara, California, USA). Ultimately, the library underwent sequencing on an Illumina NovaSeq 6000 platform (Illumina, San Diego, California, USA), yielding 250 bp paired-end reads.

### 16S rRNA gene sequencing data preprocessing

The initial phase involved the processing of raw paired-end reads from each sample using FastQC software (https://www.bioinformatics.babraham.ac.uk/projects/fastqc/) to eliminate invalid or low-quality data. Measures included removing reads with a mean sequence quality (Phred score) below 20 and reads featuring more than 3 N bases. Subsequently, Cutadapt software [22] was employed to trim the adapter sequences of each read and to deconvolute the sequences into sample bins. The ensuing stage entailed merging the trimmed paired-end reads into tags via FLASH software (v1.2.7) [23]. Then, the DADA2 module within QIIME2 software was employed for dereplication and denoising [24] and to generate amplicon sequence variant (ASV) as representatives, with an abundance threshold greater than five. Taxonomic assignments for the representative sequences of each ASV were determined by aligning the reads to the Silva database (Release 138) [25] using QIIME2. The ASV number of each sample was rarefied to 24,000 using the rarefy function from the R 'vegan' package (v2.6.4) [26]. The normalization of absolute ASV abundance was conducted using the read count from the sample with the fewest reads (39,194) in the processed data. Furthermore, only ASVs with an average relative abundance exceeding 0.01% and detected in at least 50% of samples were selected for futher analysis.

### Metagenomic library and sequencing

In addition to the 16S rRNA gene-based microbial analysis, a subset of the cohort underwent metagenomic analysis. Specifically, 29 piglet rectal swab samples from 10 sows, as well as 10 rectal swab samples and 10 vaginal swab samples from sows, were randomly chosen for metagenomic library construction, followed by 150 bp paired-end sequencing on the NovaSeq 6000 platform (Illumina, San Diego, California, USA). The bioinformatic workflow to create a gene catalog unfolded as follows. Initial trimming of adapter sequences and removal of low-quality reads from raw data were executed using fastp software with default parameters [27]. Trimmed reads were then aligned to the pig reference genome (*Sscrofa11.1*) via BWA software [28], with host genome contamination eliminated using SAMtools software [29]. In this study, metagenome assembly was pursued through two distinct approaches to identify the more effective strategy. The first strategy and parameters were informed by the study of Chen et al. [30], characterized by individual independent assembly coupled with unmapping reads mixed assembly. The second strategy and parameters were influenced by the study of Delgado et al. [31], involving individual independent assembly and mixed assembly of reads across all samples. Following assembly, the contig sequences from the aforementioned strategies were subjected to gene prediction via Prodigal software [32], with retention of only complete genes containing both start and stop codons. To enhance microbial gene coverage, the complete genes from the two strategies were amalgamated with 7,685,872 nonredundant genes from the study by Xiao et al. [33]. This approach not only contributes to a broader representation of microbial genes but also facilitates the identification of low-abundance microorganisms, which might face challenges in fully assembling their genes within our sequencing data. Redundant genes were removed through CD-HIT [34] to construct a combined gene catalog using the parameters -c 0.90 -s 0.8 -n 5 -M 80000. The ‘c’ parameter established a 90% sequence identity threshold for clustering, grouping sequences with similarity exceeding 90%. The ‘s’ parameter accommodated sequences with length differences up to 80% for clustering. The ‘n’ parameter determined the word size for the initial sequence comparison. The ‘M’ parameter limited memory usage during clustering to 80,000 Mb. Ultimately, the second strategy emerged as superior in terms of contig number, average contig length, complete gene count, average gene length, and non-redundant gene number (Additional file 1: Table S1); thus, it was adopted for subsequent analysis.

Building upon the protein sequences of the final gene catalog, we pursued species annotation and functional annotation as per the pipeline described in the study by Chen et al. [5]. In essence, all genes were aligned to the UniProt TrEMBL database (https://www.uniprot.org/statistics/TrEMBL) to isolate genes affiliated with bacteria, viruses, and archaea. Diamond software (v0.9.21.122) was employed for the mapping process, utilizing e-values ≤ 1e −5 and taxonomic classification based on LCA algorithms. Functional annotation was grounded in the Kyoto Encyclopedia of Genes and Genomes (KEGG) and Carbohydrate-active enzymes (CAZy). The gene catalog was aligned with clean reads from each sample using BWA-MEM [28], with the resultant alignments converted to BAM format using SAMtools software [29]. FeatureCounts within the Subread software suite facilitated the quantification of reads that were successfully assigned to genes, taxa, and functional terms [35]. Trimmed mean of m-values normalization was applied to ensure the equitable representation of gene, taxa, and function term abundances.

### Sequencing data statistics and analysis

To assess community richness and evenness, alpha diversity indices, namely, Shannon and Chao1, were calculated utilizing the "vegan" package (v2.6.4) within R software (v4.1.3). These indices were computed based on the abundance matrices of ASVs for 16S rRNA gene data and species abundance for metagenomic data. The Bray‒Curtis distance matrices, generated using the "vegan" package, were employed to quantify microbiota compositional differences across samples from different developmental stages of the offspring, as well as fecal and vaginal samples from the sows. Visual representation of the results was achieved through non-metric multidimensional scaling (NMDS) performed in R. The analysis of similarities (ANOSIM) was conducted using the "vegan" package to ascertain significant variations between groups, employing 9,999 permutations. Additionally, the impact of factors such as age, sow identification, sex, individual, and pen information on the variance of microbial community composition within samples was evaluated using permutational multivariate analysis of variance (PERMANOVA). This analysis was carried out on metagenomic data using the "adonis2" function from the "vegan" package. To identify biomarkers at different taxonomic levels (phylum, genus, and species), the "microeco" package was employed to perform LDA effect size (LEfSe) analysis based on both 16S rRNA gene sequencing and metagenomic data. For weight gain data, a multiple linear regression was conducted, and residual correction was performed using the "lm()" function. Associations between microbiota composition at various taxonomic levels and corrected weight gain at the weaning period of each piglet were determined using the Pearson correlation method. The origin of colonized microbiota at each time point during piglet early life was determined using the FEAST package [36] in R. Interaction networks within each group were analyzed and visualized using the "ggClusterNet" package in R, employing the "model_maptree2" layout algorithm. The network's robustness, which signifies its resistance to interference, was assessed at the species level [37]. The stability and robustness of the gut microbiota when faced with perturbations heavily rely on its functional redundancy (FR) [38]. Ecosystems with elevated FR levels tend to exhibit greater resistance to the introduction of new species, as the newly added species are inclined to possess functional similarities with the existing ones [39]. In this investigation, we quantified the colonization resistance of the gut microbiota at distinct time points by gauging the FR value. This assessment was carried out through the application of the "uniqueness" function within the "adiv" package [40], utilizing the abundance matrix of carbohydrate metabolism genes. The species functional dissimilarity matrix utilized in the "uniqueness" function was generated following the methodology outlined in the study by Mouillot et al. [41]. For statistical comparisons, the Wilcoxon rank sum test was applied to assess significance in alpha diversity and taxonomy abundance between different sample groups. The false discovery rate (FDR) method was employed for multiple comparison correction.

## Results

### Sequencing data summary

To determine the optimal time point for the introduction of external probiotics during early-life pig development, we conducted a longitudinal gut microbiota study encompassing three crucial time points prior to piglet weaning. This investigation involved the analysis of 235 samples through 16S rRNA gene sequencing to unravel the longitudinal shifts in microbial composition. Additionally, 107 samples were subjected to metagenomic sequencing to uncover longitudinal alterations in both microbial composition and function during piglet early life (Fig. 1a). In 16S rRNA gene sequencing, a total of 24,434,249 raw reads were generated, resulting in 21,030,230 clean reads after quality control and merging. On average, each sample yielded approximately 89,490 reads (mean ± standard deviation: 89,490 ± 11,613.2). The range of sequence depths across samples spanned from 22,087 to 60,996X (Additional file 1: Table S2). Moreover, 18,610 ASVs were clustered and annotated into 31 phyla, 67 classes, 159 orders, 254 families, and 508 genera. Metagenomic samples were sequenced to an average depth of 54.5 million 150 bp reads, and an average of 30.6 million reads remained after eliminating host contamination (Additional file 1: Table S3). Upon amalgamation with a publicly available gene catalog [27] and subsequent redundancy removal, a total of 4,810,232 and 5,025,530 genes were retained from two distinct assembly strategies (methods). The public data contributed 2,805,306 and 2,797,396 of these genes, respectively. This observation suggests that the strategy involving individual independent assembly combined with the assembly of mixed sample reads may optimize the utilization of metagenomic sequencing data compared to the common approach of individual independent assembly combined with the assembly of unmapped reads. Subsequently, the gene sequences from the second strategy were assigned to 88 phyla, 109 classes, 203 orders, 375 families, 1,183 genera, and 5,974 species. At the phylum level, metagenomic analysis revealed nearly three times as many phyla compared to 16S rRNA gene sequencing. Nonetheless, within the metagenomic data, the combined abundance of those phyla not covered by the 16S results accounted for approximately 6% of the overall metagenomic abundance. Among these, 4 were archaeal phyla and 24 were eukaryotic phyla (Additional file 1: Table S4). This observation underscores the capability of metagenomic sequencing to detect a broader spectrum of species within microbial communities, including archaea and fungi, beyond the scope of 16S rRNA gene sequencing.

### Dynamics of the swine gut microbial composition in early life

In the initial analysis, comparing microbial composition in terms of alpha diversity revealed significant trends. The Chao1 index, indicating microbial richness, demonstrated a notably higher value at D21 in comparison to both D0 and D10, as evident from the abundance of ASVs obtained through 16S rRNA gene sequencing (Fig. 1b). Furthermore, the microbiota at D0 exhibited a lower Shannon index, a measure of microbial evenness, compared to the other two time points (Fig. 1c). These patterns remained consistent when Chao1 and Shannon indices were calculated using species abundance data generated from metagenomic sequencing, as seen in Additional file 2: Fig. S1. Notably, in vaginal samples, differences in Chao1 indices obtained from metagenomic sequencing diverged from those derived from 16S rRNA gene sequencing, likely influenced by the effective sequencing data amount. Next, we explored variations in microbiota composition across different time points by leveraging the Bray‒Curtis distance metric to quantify dissimilarities between pairs of samples at both the ASV and species levels. This beta-diversity measure provided insight into the dissimilarity between samples. Visual representation of the resulting distance matrix was achieved through NMDS. There was distinct separation between fecal and vaginal samples from sows and offspring fecal samples. Additionally, offspring fecal samples exhibited a discernible transition pattern aligning with their developmental stages during lactation (Fig. 1d and Additional file 2: Fig. S2). Analysis of similarities reinforced these observations, indicating statistically significant differences in bacterial communities based on origin and developmental time (*P* < 0.001) (Fig. 1d and Additional file 2: Fig. S3).

Moreover, we further compared the alpha diversity and beta diversity of microbiota obtained under 16S rRNA gene sequencing and metagenomic sequencing for the same samples at the genus level. Except to sow vagina samples, other samples obtained higher species richness (Chao1) after metagenomic sequencing, but the obtained species evenness (Shannon) was lower than the latter (Additional file 2: Fig. S4). The occurrence of this situation in vaginal samples may be due to the relatively serious contamination of the host genome, resulting in insufficient metagenomic detection capabilities (Additional file 1: Table S3). On beta diversity, the D0 samples under metagenomic sequencing were closer to the sow vaginal samples, while the D0 samples under 16S sequencing shows that the D0 samples is closer to the D10 samples. However, based on these two sequencing methods, when comparing the distances between samples at the three time points of D0, D10 and D21, both methods showed the characteristics of gradual changes in the microbial composition of the samples over time (Additional file 2: Fig. S5).

Building upon these observations, we employed 16S rRNA gene sequencing analysis to delve into taxonomic composition at both the phylum and genus levels. Meanwhile, the results from metagenomic sequencing were utilized to characterize taxonomic composition at the species level.

### Common and age-associated microbes in early life

**Fig. 2:**
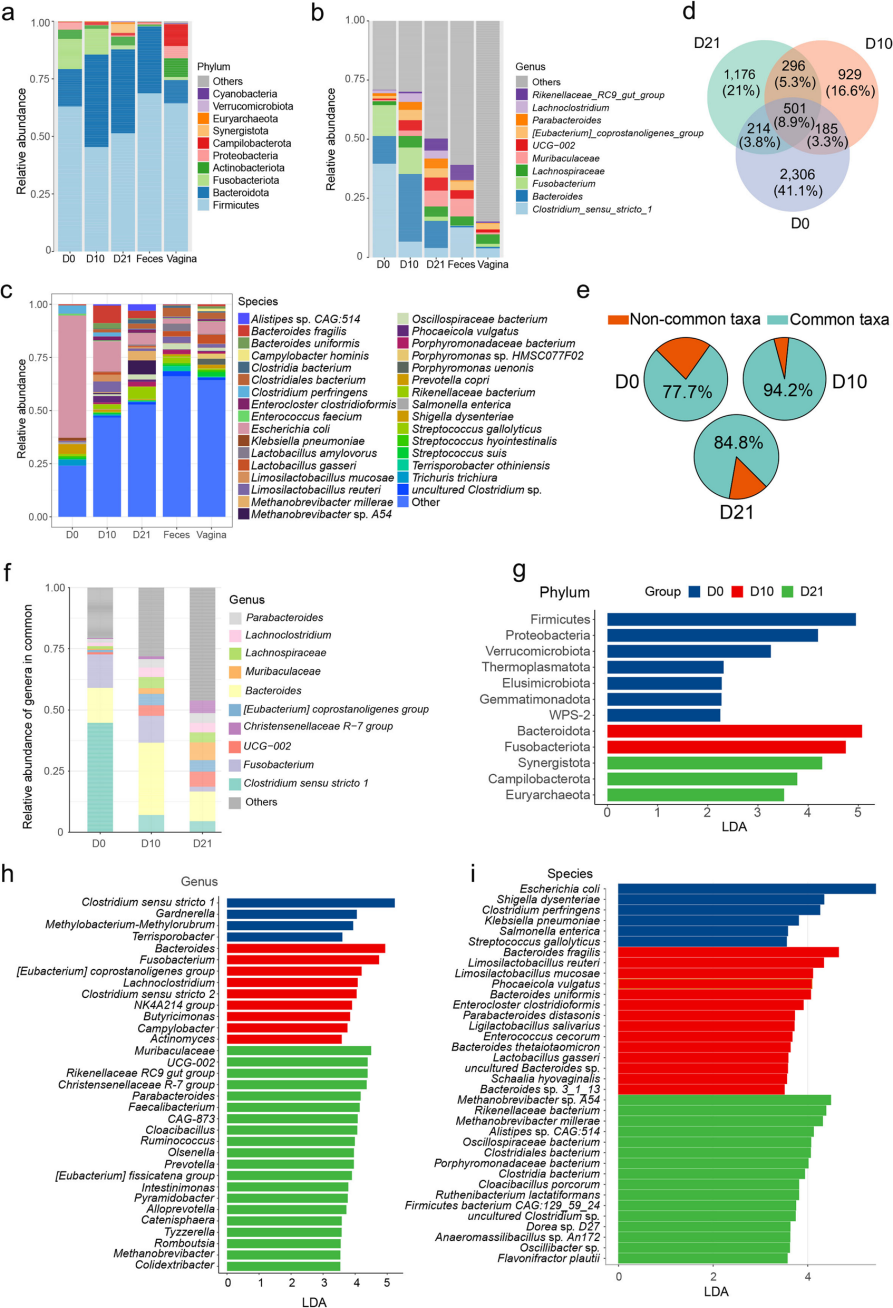
The composition analyses of the fecal microbial communities between groups that derived from 16S rRNA gene sequencing and metagenomic sequencing data. **a** Microbial composition at the phylum level. **b** Microbial composition at the genus level. **c** Microbial composition at the species level. The groups are represented along the horizontal axis, and relative abundance is denoted by the stack bar charts. **d** The overlap and unique ASVs observed at three time points (D0, D10, and D21). Each circle represents a specific time point, and the numbers inside the circles indicate the number of ASVs unique to that time point. The overlapping areas between the circles indicate the number of shared ASVs among the time points. The proportion in square brackets below the number is the ratio of the number in a area to the total ASVs number. **e** The abundance proportion of ASVs shared by the three time points at each time point by the pie charts. **f** Relative abundance of genera in common. The time pionts are represented along the horizontal axis, and relative abundance is denoted by the stack bar charts. **g** LEfSe identified significantly different bacterial taxa at phylum level according to the relative abundance among the three time points. **h** LEfSe identified significantly different bacterial taxa at genus level. **i** LEfSe identified significantly different bacterial taxa at species level. Taxa in this graph were statistically significant (*P* < 0.05) and had an LDA Score > 2.5 (phylum level) or 3.5 (genus and species level), which was considered a significant effect size

Before weaning, the fecal microbiota of piglets exhibited dominant phyla, including Firmicutes, Bacteroidota, Proteobacteria, and Actinobacteria, based on their relative abundance. Notably, Proteobacteria displayed the highest abundance at D0, gradually decreasing over time. In contrast, the abundance of Bacteroidota notably increased after birth. Furthermore, Synergistota and Euryarchaeota contributed a significant portion of the fecal microbiota at D21 (Fig. 2a and Additional file 2: Fig. S6). At the genus level, *Clostridium *sensu stricto 1, *Bacteroides*, and *Fusobacterium* were the predominant genera observed at both D0 and D10, although their relative abundance rankings varied. As development progressed, the relative abundance of genera became more evenly distributed, resembling the proportions seen in the fecal microbial composition of adult sows at D21 (Fig. 2b). At the species level, based on metagenomic sequencing data, the dominant taxa exhibited variation across each developmental stage. The D0 fecal samples showed a notable prevalence of *Escherichia coli* and *Clostridium perfringens*. *Bacteroides fragilis* and *Limosilactobacillus reuteri* were highly colonized at D10. Upon reaching D21, the abundance of microbes associated with fermentation function, such as *Methanobrevibacter* sp. *A54* and *Methanobrevibacter millerae*, increased significantly. This suggests the early-life potential of the piglet intestinal microbiota for polysaccharide degradation (Fig. 2c). Further analysis of the ASV microbial composition across the three time points revealed that only 501 ASVs (9%) were consistently present throughout all three stages. Intriguingly, the D0 microbiota demonstrated the highest proportion of age-associated ASVs, whereas the D10 microbiota had the lowest proportion of such ASVs (Fig. 2d). Notably, a substantial portion of the age-associated microbes in D0 exhibited transient and low abundance. Additionally, the ASVs shared across all three time points accounted for an average of 85.5% of the total microbial community abundance (Fig. 2e). The subsequent development of the microbiota demonstrated the changing proportion of these pivotal microbes over time (Fig. 2f). Relative abundances of taxa at different time points were compared using LEfSe analysis to identify representative taxa during each time (LDA ≥ 3.5). At the phylum level, the D0 microbiota exhibited higher relative abundances of Firmicutes and Proteobacteria. In contrast, Bacteroidota and Fusobacteriota dominated the D10 fecal microbial community. At D21, Synergistota, Euryarchaeota, and Campilobacterota displayed increased colonization (Fig. 2g). At the genus level, 4, 9, and 20 genera with advantageous proportions were identified in D0, D10, and D21, respectively. *Clostridium *sensu stricto 1, *Bacteroides*, and *Muribaculaceae* were the most representative genera of D0, D10, and D21, respectively (Fig. 2h). In terms of species-level analysis, 36 species with differing abundances across the three time points were identified (Fig. 2i). Notably, the dominant species at each time point were also abundant during that specific time (Additional file 2: Fig. S7). Additionally, substantial within-group variation in abundance was observed for *Lactobacillus gasseri* across all three time points (Additional file 1: Table S4). At D0, the majority of identified species biomarkers in piglet fecal microbiota were facultative anaerobes, including well-known examples such as *Escherichia coli*, *Shigella dysenteriae*, and *Klebsiella pneumoniae*. Conversely, biomarker taxa at D10 and D21 mainly belonged to anaerobic species, such as *Limosilactobacillus reuteri*, *Phocaeicola vulgatus*, *Clostridiales bacterium*, and *Porphyromonadaceae bacterium* (Additional file 1: Table S5). Moreover, we conducted a comprehensive comparison of the outcomes derived from LEfSe analysis at the genus level using both 16S rRNA gene sequencing and metagenomic sequencing data on same samples. Through the utilization of metagenomic and 16S rRNA gene sequencing data, we were able to identify 54 and 64 genera as distinct biomarkers across the various groups, respectively. A total of 20 genera were identified as biomarkers using both methods, with 1, 2, 6, 4, and 7 genera identified in the D0, D10, D21, sow feces, and sow vagina, respectively (Additional file 1: Table S6).

### Source of acquisition and factors that drove the intestinal microbiota

**Fig. 3:**
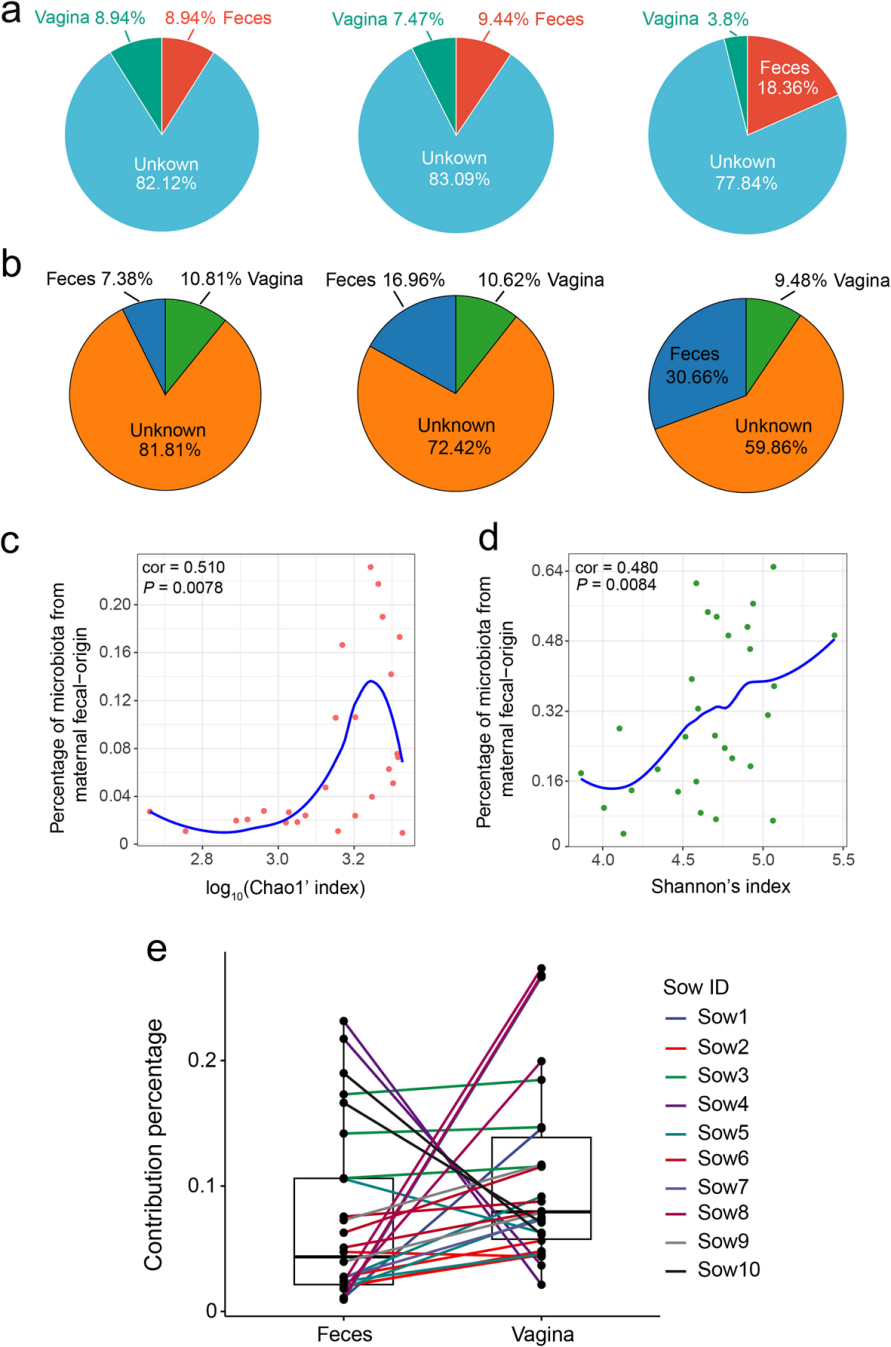
Source of piglet fecal microbiota at three sampling times in early-life. **a** Proportion of microbiota from piglet feces at different days that estimated the origin from sow feces and vagina based on 16S rRNA gene sequencing data at ASVs level. **b** Proportion of microbiota from piglet feces at different days that estimated the origin from sow feces and vagina based on metagenomic sequencing data at species level. **c** Relationship between the proportion of fecal microbiota in the microbiota of piglets and Chao1 index of microbiota of piglets at species level. **d** Relationship between the proportion of fecal microbiota in the microbiota of piglets and Shannon index of microbiota of piglets at species level. Each point represents one sample. The *X*-axis represents the log_10_(Chao index) of the sample microbiota, *Y*-axis represents the proportion of sow fecal microbiota in the microbiota of piglets. The correlation coefficient (Cor) indicates the strength of the correlation. *P*-value less than 0.05 indicates a significant relationship. **e** Paired box plots showing the proportion of sow fecal and vaginal microbiota in the microbiota of each piglet. Each point represents one sample, and lines of the same color represent full siblings

The colonization of common microbes in piglets' early life commences rapidly from birth. However, as the piglets approached weaning, significant adjustments occurred in the composition of core microbes to meet the host's physiological demands. To delve deeper into this phenomenon, we conducted further analyses to explore the origin of gut microbes in piglets and the factors influencing the shifts in microbial composition. At both the ASV and species levels, the origin tracking analysis revealed that the intestinal microbiota of piglets underwent colonization by a multitude of microbes originating from the sow's vagina and feces during the process of delivery (Fig. 3a and b). From D0 to D21, the proportion of maternal-origin microbes in piglets' intestines surged from 18% to 40%, underscoring the pivotal role of maternal microbes in shaping the early-stage intestinal microbial community structure of piglets (Fig. 3b). Furthermore, noteworthy fluctuations were observed in the proportion of maternal vaginal and fecal origin microbes within the piglets' intestines. Initially, the proportion of vaginal microbes exceeded that of fecal microbes at birth; however, by D21, the proportion of fecal-origin microbes had become several times higher than that of vaginal-origin microbes (Fig. 3b). This implies that the sow fecal microbiota may play a more significant role in shaping the intestinal microbiota at weaning compared to the sow vaginal microbiota. A substantial positive correlation was noted between the proportion of maternal fecal-origin microbes and the richness of the intestinal microbiota in piglets at birth (Fig. 3c). Additionally, these maternal fecal-origin microbes exerted a significant influence on the evenness of the intestinal microbiota in weaned piglets (Fig. 3d). Furthermore, it was evident that full-sibling piglets from the same sows exhibited greater similarity in the microbial composition originating from maternal fecal and vaginal sources at birth compared to nonfull-sibling piglets (Fig. 3e). Beyond the maternal effect, we also examined the impact of age, sex, pen (building), and individual factors on the microbial composition of piglets. The PERMANOVA analysis results revealed that age, sow, and pen were significant drivers of changes in the intestinal microbial composition of piglets (*P* < 0.05). Among these factors, age displayed the most pronounced effect (*R*^2^ = 0.066) (Table 1).

**Table 1:** Factors that can drive changes in intestinal microbial composition of piglets

Factor	Sum of squares	*R*^2^	F model	*P*r(> F)
Age	8.1	0.42	30.93	0.001
Pen	0.75	0.039	1.9	0.043
Sow	1.28	0.066	1.63	0.044
Sex	0.13	0.007	1.02	0.34
Individual	2.03	0.11	0.86	0.78
Residual	6.94	0.36		

*Pr(*> *F)* Permutational *P*-values using pseudo-F ratios

### Functional characteristics of gut microbiota in piglets’ early-life

**Fig. 4:**
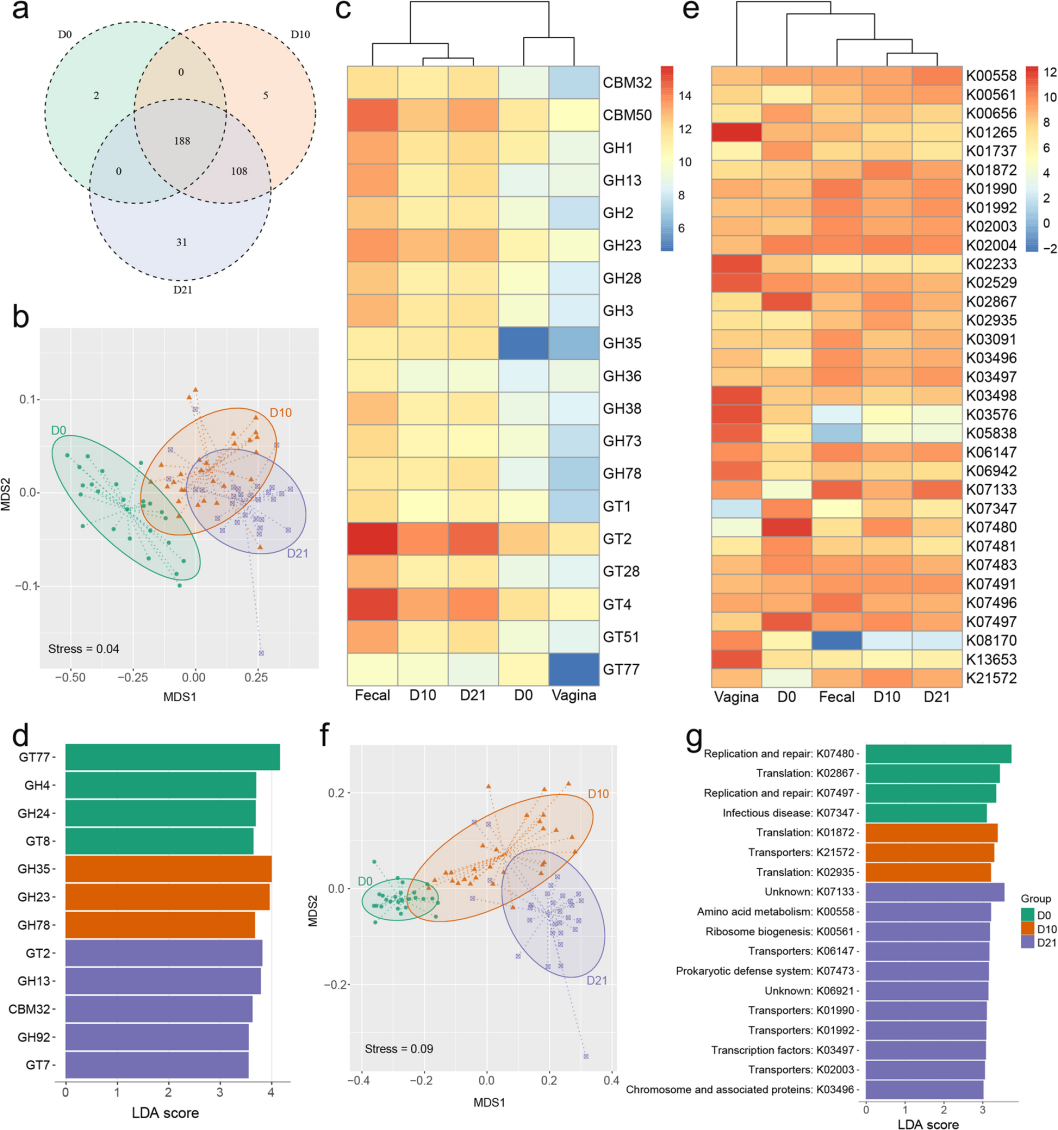
Functional annotation of the microbiota. **a** The overlap and unique carbohydrate-active enzyme (CAZy) families observed at three time points (D0, D10, and D21). Each circle represents a specific time point, and the numbers inside the circles indicate the number of CAZy family unique to that time point. The overlapping areas between the circles indicate the number of shared CAZy family among the time points. **b** NMDS plot of three time points based on the abundance of CAZy families. **c** Heatmap of the 10 most abundant (based on TMM value) CAZy families in any of the groups. Color scale shows the abundance of CAZy family within each group. Z-score, calculated with the formula *z* = (*x* − *μ*)/*σ*, where *x* is the log2 of abundance of enzymic families in each group, *μ* is the mean value of the log2 of abundance in all groups, and *σ* is the standard deviation of the log2 of abundance. **d** LEfSE analysis for CAZy families to compare the microbiota functional profiles among three time points. **e** Heatmap of the 10 most abundant (based on TMM value) KEGG Orthology (KO) in any of the groups. **f** NMDS plot of three time points based on the abundance of KOs. **g** LEfSE analysis for KOs to compare the microbiota functional profiles among three time points. Functional terms in this graph were statistically significant (*P* < 0.05) and had an LDA score > 3, which was considered a significant effect size

To gain a deeper understanding of the functional diversity within the microbiota, we employed two functional databases for gene annotation. First, we aligned sequences from the final gene catalog with protein sequences in the CAZy database, allowing us to categorize sequences into 6 enzyme classes and 510 families. Among these, 334 families were identified in the piglets' intestinal microbiota during the weaning process, including 2, 5, and 31 time-specific families at D0, D10, and D21, respectively. Similar to the taxonomic composition (Fig. 2e), a substantial proportion of enzyme family terms were shared across the three stages (Fig. 4a). At the enzyme family level, the top 10 most abundant terms in the piglet microbial community at D10 and D21 resembled those in the sow fecal samples, whereas D0 exhibited greater similarity to the sow vagina. Notably, carbohydrate-binding module family 50 (CBM50), glycoside hydrolase family 23 (GH23), glycosyltransferase family 2 (GT2) and GT4 were common high-abundance enzyme families at all three time points (Fig. 4b). Utilizing the Bray–Curtis distance, the abundances of CAZy families allowed for the grouping of samples into three clusters, each displaying distinct transitional characteristics corresponding to their developmental stages (Fig. 4c). A total of 12 CAZy families were identified as functional biomarkers across the three time points. The biomarker enzyme families at D0 encompassed galactosyltransferase and glucosidase, while those at D10 included peptidoglycan lyase and rhamnosidase. At D21, cellulose synthase and amylase were among the prominent biomarker enzyme families (Fig. 4d).

Furthermore, the gene catalog was assigned to the KEGG database, identifying 7,278 KEGG orthologs (KOs) in this study. The dominant KOs in the sow vaginal group significantly differed from those in the other groups, and the dominant KOs in the piglet fecal samples at D0 exhibited distinctions from those at the other two time points (Fig. 4e). Employing the Bray–Curtis distance, computed using the KO abundance matrix, allowed the grouping of piglet fecal samples into patterns corresponding to D0, D10, and D21 (Fig. 4f). To further differentiate the functional characteristics among the three time points, LEfSe analysis was employed. Using a cutoff LDA score = 3, we identified 18 KOs showing significant differences. These KOs primarily participated in genetic information processing, albeit with subtle differences in their specific involvement. The functional attributes at D0 were largely associated with replication and repair, as well as translation. D10 exhibited a greater association with translation and transporters, while D21 showed stronger links to transporters, ribosome biogenesis, and prokaryotic defense systems (Fig. 4g). These findings suggest that the functional properties of the gut microbiota during piglets' early life exhibit shared features but also undergo clear transitions.

### Microbial interaction and colonization resistance in early-life

**Fig. 5:**
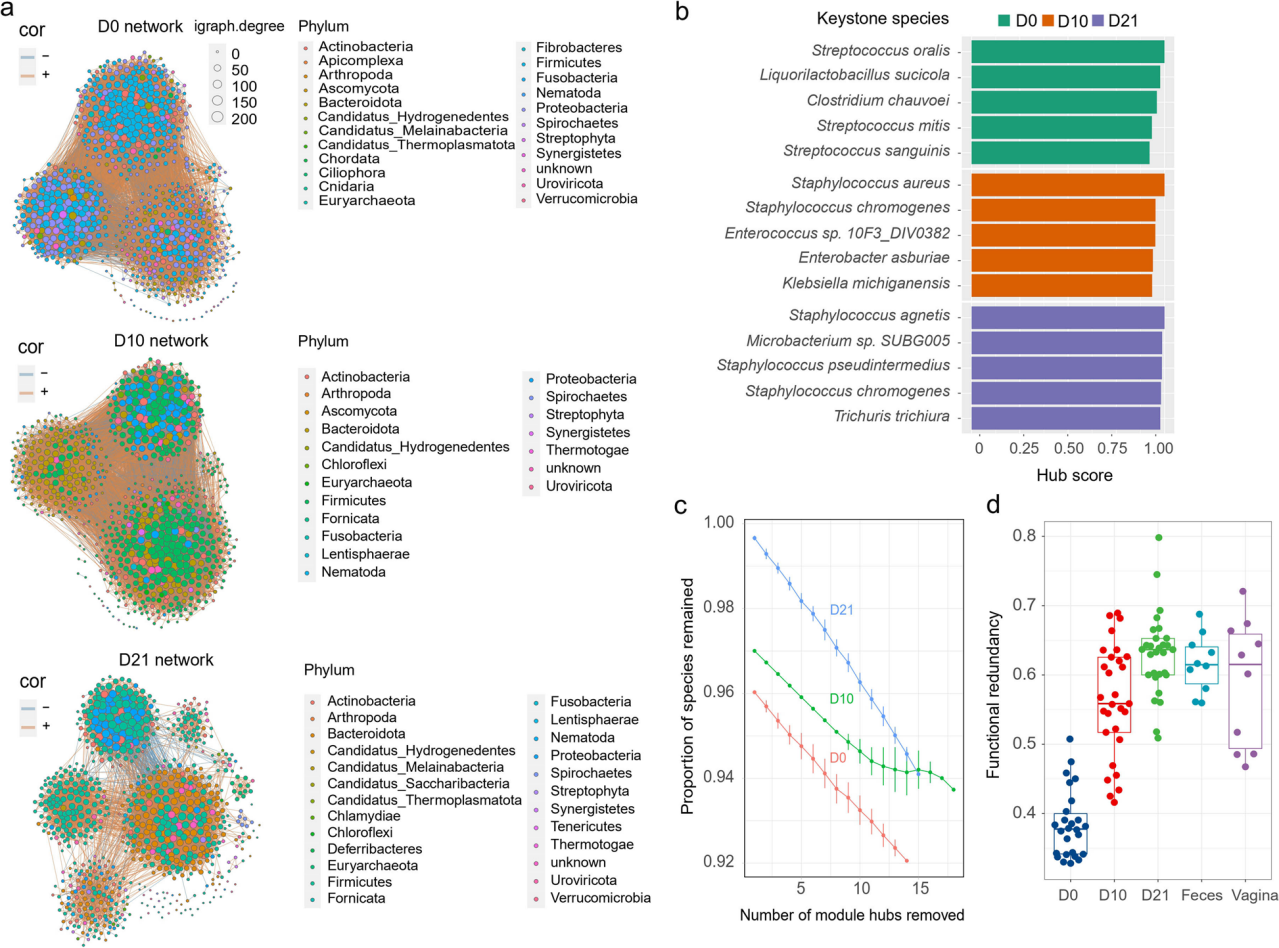
Microbial interaction network and colonization resistance of piglet's fecal microbiata at different time points. **a** The network diagram represents the microbial interaction network within the fecal microbiota of piglets at three different time points. Each point in the network corresponds to a microbial taxon, and its size represents the relative abundance of the taxon. The color of each point represents its taxonomic classification. The connections (lines) between points indicate the positive or negative correlation between taxa. Positive correlations are depicted in red, while negative correlations are depicted in blue. **b** The keystone species within the microbial interaction network at each time point. The *Y*-axis represents the names of the species, while the *X*-axis represents the corresponding hub score, which indicates the importance of the species in maintaining the network structure. **c** The line graph depicts the evaluation of network robustness by employing a random removal method of species to assess the network's ability to withstand interference. The *X*-axis represents the percentage of randomly removed microbial species, incremented by 0.05 at each step, while the *Y*-axis represents the calculated network robustness. The robustness values at different time points are represented by different colored lines, illustrating the changes in network robustness over the course of time. **d** The boxplot illustrates the variation in functional redundancy across different groups. The individual points on the plot represent each sample within the respective group, showing the specific functional redundancy value for that sample

The intricate interactions among microbes within the microbiota have a substantial impact on its stability and functionality. In this study, the microbial interaction network was utilized to visualize the relationships and characteristics of microbial interactions. At D0 and D10, the piglet fecal microbial interaction network could be classified into three major clusters, while at D21, it expanded to five major clusters. Throughout all three time points, the key components of the microbial interaction networks were the phyla Firmicutes, Proteobacteria, and Bacteroidota. The proportions of these phyla varied over time, with Firmicutes and Bacteroidota dominating at D10 and D21, while Firmicutes and Proteobacteria were prominent at D0 (Fig. 5a). Upon examining the network's internal connections, we identified keystone species within each network, which were characterized by high hub scores. At D0, three species (*Streptococcus oralis*, *Streptococcus mitis*, and *Streptococcus sanguinis*) were identified as keystone species. At D10, two species (*Staphylococcus aureus* and *Staphylococcus chromogenes*) were identified as keystone species. While at D21, three species (*Staphylococcus agnetis*, *Staphylococcus chromogenes*, and *Staphylococcus pseudintermedius*) were identified as keystone species (Fig. 5b).

To assess the network's resistance to perturbations, we calculated its robustness by systematically removing varying proportions of microbes. Intriguingly, the D21 microbial interaction network demonstrated greater robustness than D10, particularly when fewer than 15% of microbes were removed. Conversely, the D0 network exhibited relatively lower robustness (Fig. 5c). Surprisingly, despite having the highest number of subclusters, the D21 microbial interaction network displayed the fewest edges among the three time points (D0, D10, and D21) (Additional file 1: Table S7). This observation suggests that the stability of the D21 network might stem from the dominance of specific bacteria in different niches, leading to a more concise and stable network of microbial interactions.

Previous studies have indicated that more stable microbial interaction networks tend to exhibit stronger colonization resistance [42, 43]. To validate this point, we computed the functional redundancy value of the microbiota at different time points, which serves as a measure of the colonization resistance of the gut microbiota. Indeed, the gut microbiota at D21 displayed the highest functional redundancy among the three time points, followed by D10, and the least at D0 (Fig. 5d). Moreover, the microbiota at D21 also exhibited the highest richness of genes under functional classifications based on CAZy and KEGG databases compared to the other two time points (Additional file 2: Fig. S8). Considering the aforementioned insights related to microbiota niche, robustness, and functional redundancy, it can be inferred that the introduction of external probiotics during D21 will likely encounter greater colonization resistance compared to the other two time points in piglet's early life. Additionally, introducing probiotics that exhibit a competitive advantage in occupying specific niches during D0 or D10 may hold greater potential for exerting beneficial effects on host growth and health, driven by the mechanism of priority effects.

### Growth performance-associated microbes in early life

To further investigate the optimal timing for introducing external probiotics during early piglet life, we investigated the relationship between microbial composition at three distinct time points and piglet weight gain during the weaning period. Weight gain during weaning is a pivotal production trait that is influenced by various factors. To mitigate genetic effects (limited to maternal influence), sex, and housing conditions, we initially adjusted the trait phenotype of the piglets using a multiple linear regression model (Additional file 1: Table S8). With consideration of the sample size, we conducted an association analysis between the abundance of phyla and genera and the adjusted trait phenotypes, utilizing samples (*n* = 63) from 16S rRNA gene sequencing analysis.

**Fig. 6:**
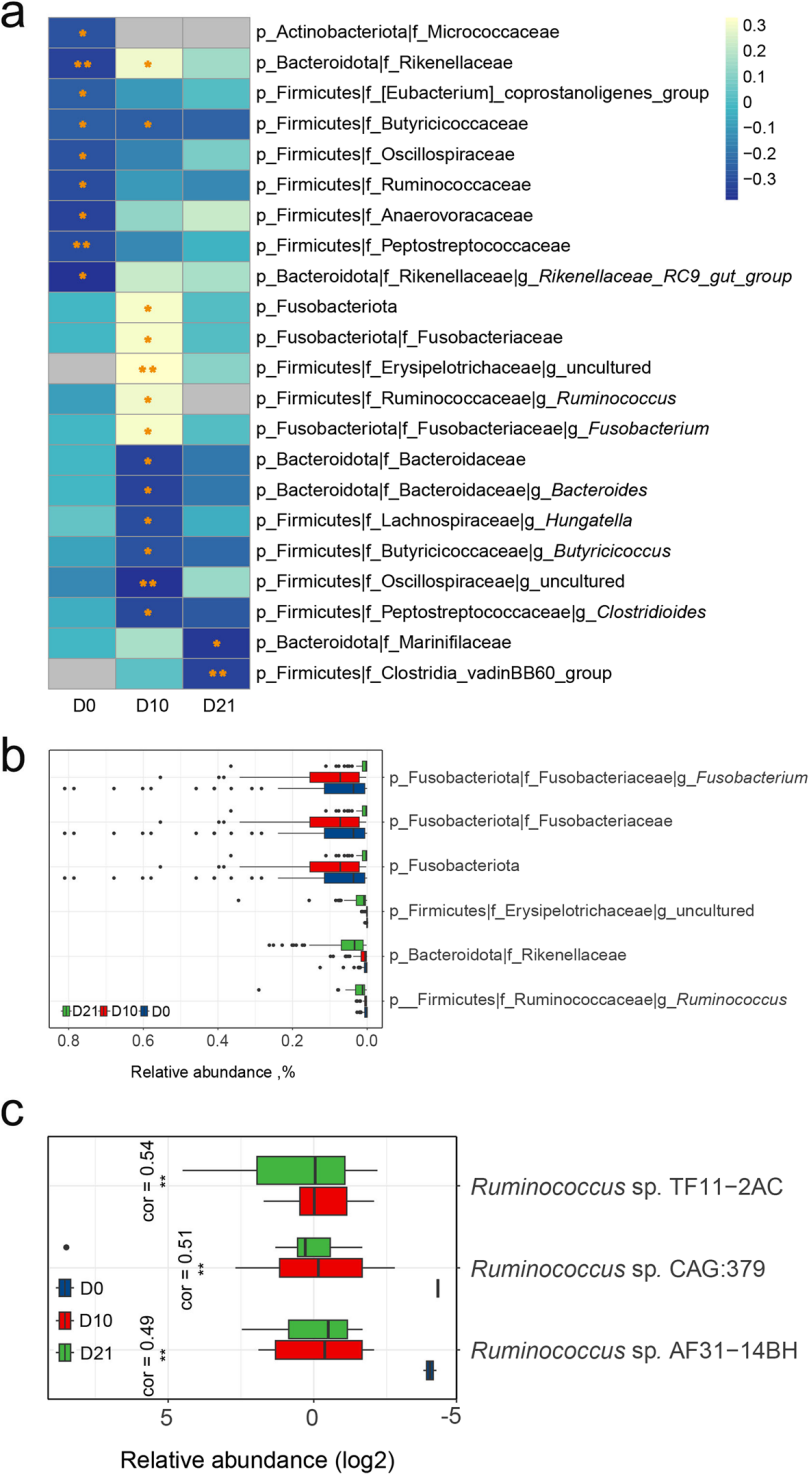
Correlation between fecal taxa abundance and weight gain during weaning. **a** Heatmap illustrating the correlation between microbial abundance and weight gain at three time points. The color gradient in the heatmap represents the correlation coefficient between microbial abundance and weight gain. The asterisks (*) on the heatmap cells indicate a significant *P*-value < 0.05 (*) or < 0.01(**). **b** Relative abundance of microbial taxa positively correlated with weight gain during weaning across three time points. **c** Boxplot illustrating the relative abundance of *Ruminococcus* species significantly positively correlated with weight gain during the weaning period at three different time points. The correlation coefficient (Cor) indicates the strength of the correlation. Asterisks (*) denote statistical significance with a *P*-value < 0.01 (**) indicating a highly significant correlation

A total of 22 taxa were identified as significantly associated with weight gain during the three stages (*P* < 0.05, FDR < 0.2) (Fig. 6a). These taxa comprised 1 phylum, 12 families, and 9 genera. Among them, 9 taxa exhibited a negative correlation with weight gain at D0, 7 taxa at D10, and 2 taxa at D21. Interestingly, all 6 taxa that showed a positive association with weight gain were identified at D10. Specifically, Fusobacteriota, Fusobacteriaceae, and *Fusobacterium*, which belong to the same branch in the phylogenetic tree, displayed a significant positive correlation with weight gain during the weaning period (Fig. 6a). Within the taxa positively correlated with weight gain, the abundance of Rikenellaceae, *Ruminococcus*, and an uncultured genus from Erysipelotrichaceae exhibited a gradual increase over the weaning process. In contrast, the abundance of Fusobacteriota, Fusobacteriaceae, and *Fusobacterium* demonstrated significant fluctuations across the three periods, peaking at D10 and experiencing a substantial decline by D21 (Fig. 6b). These taxa may be considered potential candidates for enhancing weight gain. Moreover, based on metagenomic data, we conducted a more detailed investigation into the association between bacterial species under the aforementioned genera and weight gain during weaning. Among these, only 3 species, namely *Ruminococcus* sp. TF11–2AC, *Ruminococcus* sp. CAG:379, and *Ruminococcus* sp. AF31–14BH, exhibited a significant positive correlation with weaning weight gain at D10 (*P* < 0.05, FDR < 0.2) (Fig. 6c, Additional file 2: Fig. S9). However, the relative abundance of these microbial species did not significantly increase at D21, indicating that these strains did not establish a dominant position in microbiota competition during the period from D10 to D21. In light of these findings, it can be deduced that D10 is the most appropriate time for introducing these potentially probiotic bacteria, enabling them to establish a dominant niche and potentially contribute to enhanced weight gain.

## Discussion

In contemporary pig farming practices, the integration of probiotics into pig feed has become a common strategy to enhance gut microbiota, ultimately leading to improved growth and overall phenotypic outcomes in pigs [43]. Previous research has highlighted that the early life phase presents a pivotal window for interventions aimed at influencing the mammalian gut microbiota and overall health [10, 44, 45]. The early life stage of a pig encompasses lactation and encompasses crucial events such as colostrum feeding, creep feed introduction, and weaning. While various studies have employed 16S rRNA gene sequencing to analyze the dynamic shifts in microbial composition during piglet weaning [8, 46, 47], there is a scarcity of information pertaining to functional distribution during the early stages of piglet life and the optimal time for probiotic introduction. Metagenomic sequencing not only facilitates microbial functional exploration but also offers superior sensitivity in detecting low-abundance bacteria, archaea, and fungi. For bacteria with relatively high abundance, both metagenomics and 16S sequencing can provide detection capabilities, as supported by the work of Durazzi et al. in the gut microbiota of chickens [48]. Although the current understanding of probiotics highlights their limited ability to colonize the gut, necessitating continuous dosing. Various factors affect the colonization of probiotics during gastrointestinal transit, including gastric acid, digestive enzymes, bile acids in the upper gastrointestinal tract [49]. The colonization resistance of the existing gut microbiota further hampers successful probiotic colonization. As researchers delve deeper into understanding the intricate interplay of these factors impacting probiotic colonization, there's potential for the development of novel probiotic formulations that exhibit enhanced colonization capabilities. This study delved into the longitudinal alterations in gut microbial composition, functional capacity, microbial interaction networks, and colonization resistance in the period preceding weaning, aiming to offer insights for selecting the most opportune time to introduce probiotics during piglet early life. This insight informs new probiotic candidates and innovative application strategies.

The fecal samples from the D0 group of piglets were collected within 5 h of farrowing in this investigation. The median diversity indices (Chao1 and Shannon) observed in the fecal microbiota of D0 piglets indicated the potential for external microbes to colonize the piglets' gut during this brief time frame. Source tracking analysis revealed that the vaginal and fecal microbiota of the sow played a significant role in microbial colonization during the birthing process of piglets, aligning with prior studies [46]. In addition, the microbes originating from the sow feces may play a more significant role in shaping the intestinal microbiota at weaning compared to the sow vaginal microbiota. This finding is consistent with a study conducted on human infants, which demonstrated that the contribution of the maternal vaginal microbiota to infants gradually diminishes after delivery [50]. In contrast to some previous research, the alpha diversity of the microbiota in D10 samples was higher than that of D0 samples in our study. This discrepancy could potentially be attributed to the fact that our experimental groups were exposed to creep feed, whereas the experimental pigs in Wang et al.'s study [8] were solely fed sow milk. Notably, Choudhury et al. [51] also observed a significant increase in gut microbial diversity in early-life piglets upon introduction of solid feed in comparison to those exclusively fed sow milk. However, the mechanisms underlying how creep feed introduction impacts microbial colonization in piglet guts, whether by enhancing exposure to environmental microbes or bolstering the host immune system's tolerance to a larger microbial biomass, remain to be fully understood. Further investigations are necessary to elucidate the intricate mechanisms underlying the effects of creep feed on microbial colonization in piglets' gastrointestinal tracts.

Furthermore, this study unveiled the dynamic developmental trajectory of the piglet microbiota during early life and identified taxa acting as biomarkers at distinct time points. The consistent identification of Firmicutes and Bacteroidetes, followed by Proteobacteria, as the dominant bacterial phyla in piglet fecal microbiota during early life corroborates findings from previous investigations [8, 46, 51]. This reinforces the understanding of the prevailing phyla contributing to piglet gut microbiota composition in the initial phases of development. Nonetheless, in contrast to the research conducted by Wang et al. [8] and Chen et al. [46], the current study found a notable abundance of Fusobacteriota in the D0 samples. This divergence might be attributed to variations in maternal and environmental microbes, which can modulate *Fusobacteriota* abundance in piglet fecal microbiota until weaning. At the genus and species levels, a multitude of taxa were identified as microbiota biomarkers for each specific time point. Similar to prior studies [46, 51], the majority of species biomarkers in the piglet fecal microbiota at D0 were facultative anaerobes, while the biomarker taxa at D10 and D21 were predominantly anaerobes. This observation aligns with observations in human studies, where the infant gut initially has limited oxygen and gradually transitions toward an anaerobic environment [52]. Consequently, the ability of probiotics with varying oxygen requirements to survive in piglet intestines during early life should also be taken into consideration.

In our prior research, a consistent correlation was noted between microbiota function and physiological function in different intestinal regions of pigs [53]. In the present study, we conducted further investigation into the functional shifts within the rectal microbiota of piglets at different time points in response to the substantial dietary changes occurring during early life. The functional biomarkers identified in D0 samples primarily revolved around the digestion of monosaccharides, such as lactose. However, with the introduction of creep feed, the functional biomarkers progressively shifted toward the digestion of polysaccharides, specifically cellulose. These functional shifts in the microbiota are in concurrence with the functional prediction outcomes reported by Chen et al. [46], who studied piglets without creep feed introduction at 0 d and 28 d (weaning). Consistent with the previous findings [51], the introduction of creep feed has the potential to foster the maturation of intestinal microbiota in piglets. This also aligns with a study conducted in mice, underscoring the role of bile acids in steering the maturation of newborn gut microbiota [54]. Bile acids play a central role in shaping the microbial community by stimulating the growth of bacteria involved in bile acid metabolism, while inhibiting the proliferation of bile-sensitive bacteria [55]. Notably, genera such as *Bacteroides* [56], *Lachnoclostridium* [57], and *Butyricimonas* [58] are characteristic components of the piglet microbiota at D10 and are associated with bile acid metabolism. Further exploration is required to comprehend the intricate interactions among bile acids, the microbiota, and diet during the early-life of piglet. Furthermore, this study delved into the microbial interactions and functional redundancy of piglet intestinal microbiota at different time points. Notably, the D21 samples exhibited a higher number of subclusters yet fewer edges in the microbial interaction network compared to the other two time points (D0 and D10). This disparity could be attributed to the fact that at D0 and D10, numerous microbes are vying for the same ecological niches, resulting in more pronounced interactions between microbial communities. On the other hand, the dominant bacteria at D21 successfully established themselves in diverse niches, culminating in a more succinct and effective network of microbial interactions. The greater stability and functional redundancy witnessed in the microbial interaction networks at D21 lend further support to these observations. As a consequence, the introduction of probiotics that compete for priority niches during D0 or D10 might hold greater potential for exerting beneficial effects on host growth and health. These findings underscore the importance of considering both timing and microbial interactions when introducing probiotics during piglets' early life.

To further elucidate the optimal timeframe for introducing external probiotics during early life, we investigated the correlation between microbial composition at three distinct time points and piglet weight gain, a pivotal metric of growth performance during weaning. Notably, three bacterial species under the *Ruminococcus* genus at D10 emerged as potential candidates for augmenting weight gain, a critical indicator of growth performance during piglet weaning. *Ruminococcus* sp. TF11–2AC is predicted to generate short-chain fatty acids [59], plays a key role in various physiological processes encompassing energy metabolism and gut health [60]. Genome analysis of *Ruminococcus* sp. CAG:379 revealed a gene encoding a protein akin to the ScaK scaffoldin, which is pivotal in harboring enzymes linked to cellular structural component metabolism, such as peptidoglycan [61]. This finding hints at the possible involvement of *Ruminococcus* sp. CAG:379 in breaking down and utilizing complex polysaccharides within the gut. *Ruminococcus* sp. AF31–14BH, previously linked to the finishing weight of Diannan small ear pigs in the study by Lan et al. [62], offers insight into its potential role in influencing piglet weight gain during weaning. Interestingly, a different prior study showed a robust correlation between the weaning weight of piglets and overall post-weaning production performance [63]. In addition, *Fusobacterium* was also found to be associated with weight gain during weaning in this study, but no microorganisms were found to be significantly associated with weaning weight gain at the species level. While numerous species within the *Fusobacterium* genus are known as opportunistic pathogens in the human oral cavity and intestines [64], it is crucial to acknowledge the possibility that this genus could encompass members with yet-to-be-discovered diverse functions. However, due to the limitations of detection technology, we have not been able to find these potentially beneficial species. In addition, the abundance of *Fusobacterium* may also have an impact on the role it plays in the host gut. In the studies conducted by Dahlstrand et al. [65] and Larsen et al. [66], it was confirmed that Fusobacteriota possesses the capability to metabolize carbohydrates, resulting in the production of short-chain fatty acids, which were thought to be related to human obesity in previous studies [67]. These studies suggest that Fusobacteriota have the potential to influence host body weight. Further research is needed to elucidate the mechanisms behind this observation and to determine which bacteria have the greater influence on weight gain during weaning. Given the far-reaching impact of early-life microbial colonization on host health and growth, delving into whether the effects of weaning weight on post-weaning growth phenotype are mediated by early-life microbial composition and functions becomes a critical area for further exploration. However, according to the outcomes of this study, the optimal time for introducing external probiotics may be as creep feed is introduced (at D10 in this study). Furthermore, considering that it is challenging to precisely measure the colonization resistance of probiotics under the influence of their own characteristics, future research in this domain should be intensified to facilitate the tailored application of probiotics.

While our study yields insights into the gut microbiota dynamics of piglets during their initial stages, there are certain limitations to this study. A notable limitation pertains to the absence of gut microbiota samples at additional time points, particularly post-weaning samples. These could provide insights into the trajectory of microbiota maturation. For a comprehensive understanding of colonization resistance across the entire life span of pigs, future investigations should encompass a broader range of time points. This would enable a more precise understanding of the colonization resistance dynamics at each developmental stage. In addition, collecting a comprehensive array of environmental samples during piglet growth can serve as a valuable reference for studying environmental microorganisms. This approach facilitates a more precise assessment of the proportional impact of sow microorganisms on piglet intestinal microbiota. Moreover, this study also did not comprehensively collect samples from sows, which could potentially impact the assessment of microbial acquisition sources in piglets. This limitation may have led to an underestimation of the contribution of maternal microorganisms at birth. Furthermore, the present challenge of isolating and cultivating candidate strains associated with weight gain during weaning obstructs in-depth investigations into their colonization effects and host advantages across various time points. Notwithstanding these limitations, this study has effectively pinpointed potential candidates at the species level within the fecal microbiota. These potentially probiotic bacteria serve as a starting point for future research efforts aimed at enhancing piglet weight gain during the early stages of life. By extending these findings, forthcoming studies can delve deeper into the colonization effects and potential benefits of these candidate microbes. While recognizing the imperative for further exploration, it is important to underscore that our study encompasses three pivotal events in piglet early life, providing invaluable insights that can guide forthcoming research endeavors aimed at pinpointing the most opportune moment for introducing probiotics to piglets. By building upon these findings, we can advance our comprehension and formulate efficacious strategies to optimize piglet gut microbiota, thereby enhancing growth and overall health.

## Conclusions

In conclusion, this study has illuminated distinct shifts in intestinal microbial diversity, composition, and function at three pivotal time points during the early life of piglets. The fecal and vaginal microbiota from sows have been identified as significant sources of the piglets' gut microbiota. Notably, the microbes originating from the sow feces may play a more significant role in shaping the intestinal microbiota at weaning compared to the sow vaginal microbiota. The microbial interaction network at D21 showed a more concise and efficient network structure, accompanied by enhanced colonization resistance, in contrast to the microbiota at D0 and D10. Furthermore, three specific strains of *Ruminococcus* sp. identified at D10 have emerged as potential contenders for enhancing weight gain during the weaning period. Taking into consideration the physiological attributes and dietary needs of piglets, the most favorable time for introducing probiotics appears to be at D10 in early life. These discoveries contribute to a deeper comprehension of the early-life microbiota in piglets, shedding light on species associated with weight gain and offering valuable insights for future probiotic interventions.

### Supplementary Information

**Additional file 1: Table S1.** Comparison of metagenomic assembly results between two methods that were described in method section. **Table S2.** The statistics of 16S rRNA gene sequencing data. **Table S3.** The statistics of metagenome sequencing data. **Table S4.** The average abundance of biomarker species at each time point. **Table S5.** The relative abundance and characteric of phyla that were only dected in metagenomic sequencing data. **Table S6.** The biomarker genera that were identified under 16S rRNA gene sequencing and metagenomic data. **Table S7.** Attributes of microbial interaction networks at three time points. **Table S8.** Phenotype and meta information of experimental animal in this study.

**Additional file 2: Fig. S1.** Alpha diversity of microbial community among different groups based on metagenomic sequencing data. **Fig. S2.** NMDS plot according sample group based on the abundance of species. **Fig. S3.** The mean of ranked dissimilarities between groups to the mean of ranked dissimilarities within groups. **Fig. S4.** The alpha diversity of microbial samples under both 16S rRNA gene sequencing and metagenomic sequencing. **Fig. S5.** The beta diversity of microbial samples under both 16S rRNA gene sequencing and metagenomic sequencing. **Fig. S6.** Microbial composition at the phylym level that calculated by metagenomic sequenceing data. **Fig. S7.** The abundance of species that were specific biomarkers taxa at each time points. **Fig. S8.** Richness of functional gene in microbial samples at three time points. **Fig. S9.** Distribution of *Ruminococcus* in microbial samples at three time points.

## Data Availability

The sequencing data in this study were submitted to the NCBI’s Sequence Read Archive (SRA) database under the accession ID PRJNA985145. Publicly available datasets were analyzed in this study can be found at http://gigadb.org/dataset/view/id/100187/token/F4CDHYruxob.

## Abbreviations

ANOSIM: Analysis of similarities
ASVs: Amplicon sequence variants
CAZy: Carbohydrate-active enzyme
FDR: False discovery rate
FR: Functional redundancy
KEGG: Kyoto Encyclopedia of Genes and Genomes
KOs: KEGG orthologs
LEfSe: LDA Effect Size
NMDS: Non-metric multidimensional scaling
PERMANOVA: Permutational multivariate analysis of variance
TMM: Trimmed mean of m-values
