# Supplementary figures and images for: Composition, function, and timing: exploring the early-life gut microbiota in piglets for probiotic interventions
Source: J Anim Sci Biotechnol. 2023 Nov 13;14:143. doi: 10.1186/s40104-023-00943-z (PMC10641937; doi:10.1186/s40104-023-00943-z)

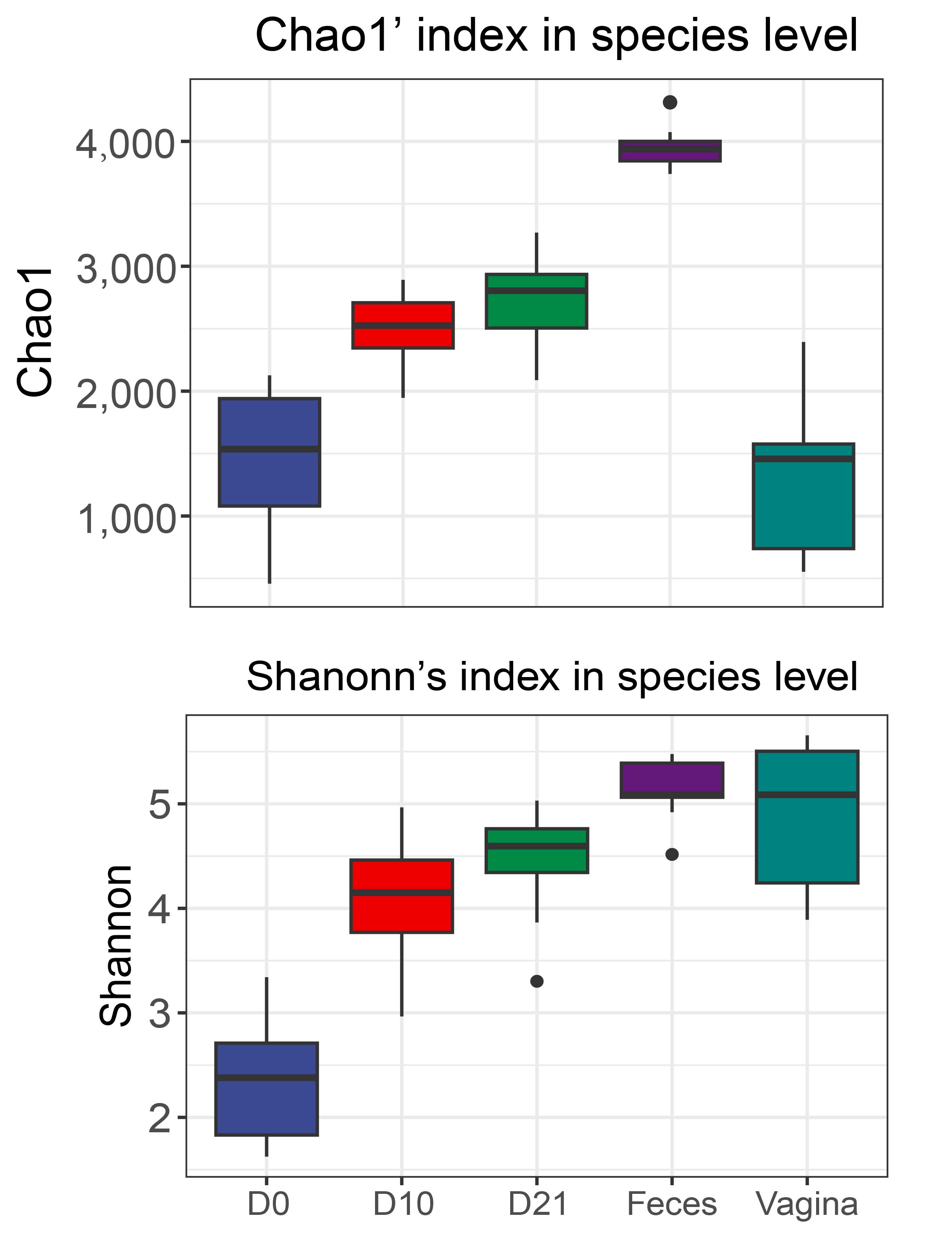

Supplement: Supplementary file 2 — Additional file 2: Fig. S1. Alpha diversity of microbial community among different groups based on metagenomic sequencing data. Fig. S2. NMDS plot according sample group based on the abundance of species. Fig. S3. The mean of ranked dissimilarities between groups to the mean of ranked dissimilarities within groups. Fig. S4. The alpha diversity of microbial samples under both 16S rRNA gene sequencing and metagenomic sequencing. Fig. S5. The beta diversity of microbial samples under both 16S rRNA gene sequencing and metagenomic sequencing. Fig. S6. Microbial composition at the phylym level that calculated by metagenomic sequenceing data. Fig. S7. The abundance of species that were specific biomarkers taxa at each time points. Fig. S8. Richness of functional gene in microbial samples at three time points. Fig. S9. Distribution of Ruminococcus in microbial samples at three time points. [file 40104_2023_943_MOESM2_ESM.zip › Figure S1.jpg]

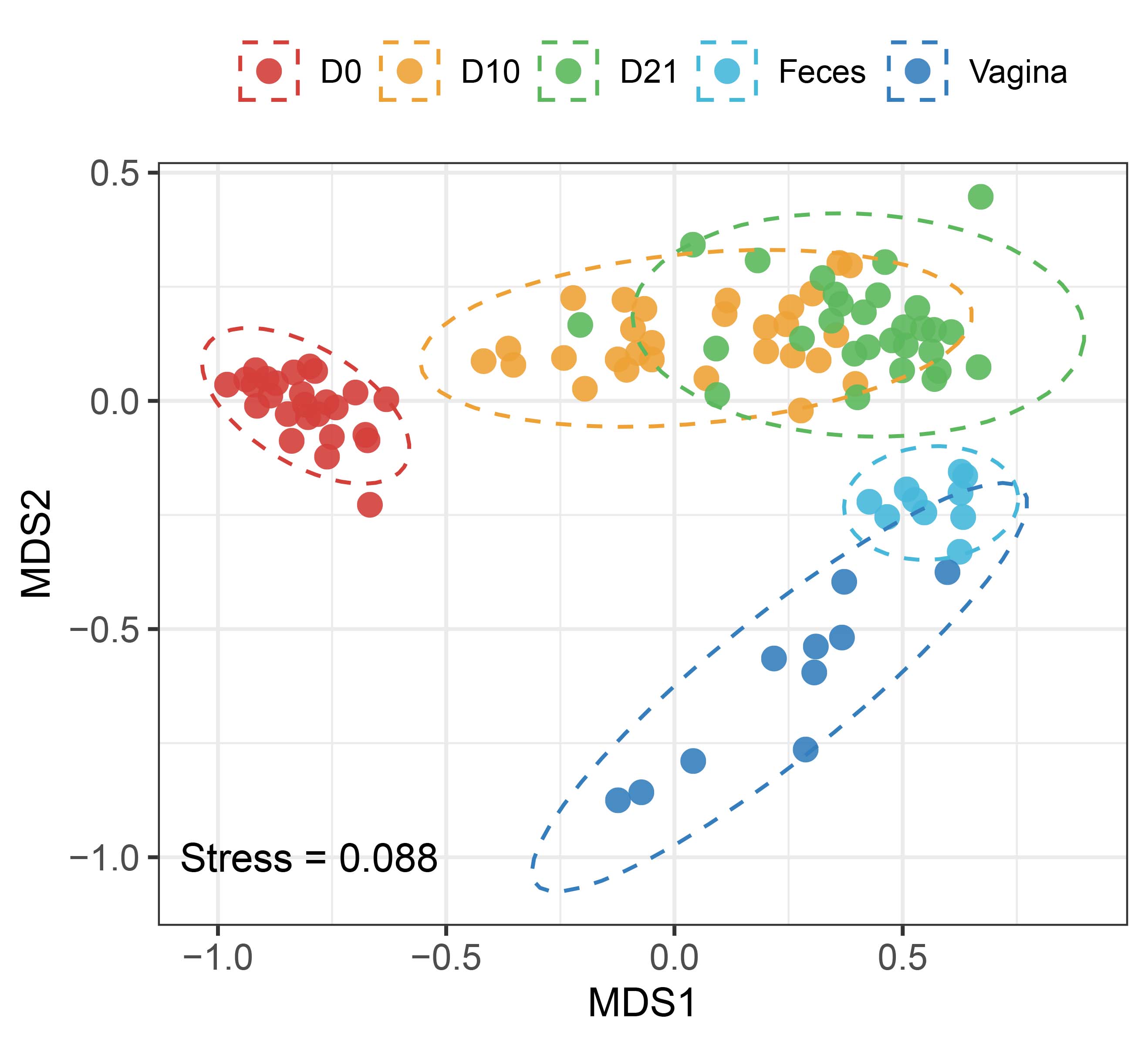

Supplement: Supplementary file 2 — Additional file 2: Fig. S1. Alpha diversity of microbial community among different groups based on metagenomic sequencing data. Fig. S2. NMDS plot according sample group based on the abundance of species. Fig. S3. The mean of ranked dissimilarities between groups to the mean of ranked dissimilarities within groups. Fig. S4. The alpha diversity of microbial samples under both 16S rRNA gene sequencing and metagenomic sequencing. Fig. S5. The beta diversity of microbial samples under both 16S rRNA gene sequencing and metagenomic sequencing. Fig. S6. Microbial composition at the phylym level that calculated by metagenomic sequenceing data. Fig. S7. The abundance of species that were specific biomarkers taxa at each time points. Fig. S8. Richness of functional gene in microbial samples at three time points. Fig. S9. Distribution of Ruminococcus in microbial samples at three time points. [file 40104_2023_943_MOESM2_ESM.zip › Figure S2.jpg]

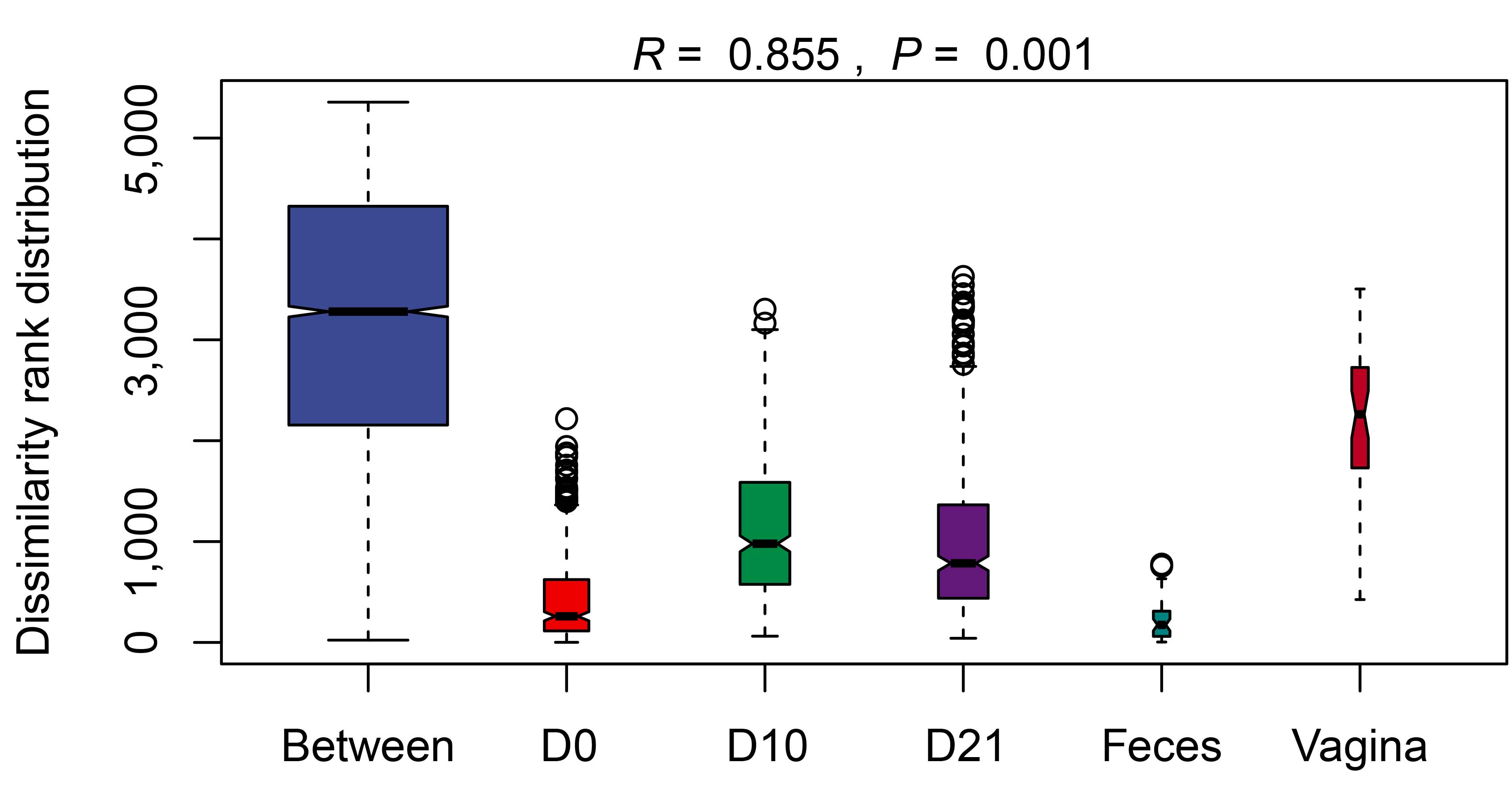

Supplement: Supplementary file 2 — Additional file 2: Fig. S1. Alpha diversity of microbial community among different groups based on metagenomic sequencing data. Fig. S2. NMDS plot according sample group based on the abundance of species. Fig. S3. The mean of ranked dissimilarities between groups to the mean of ranked dissimilarities within groups. Fig. S4. The alpha diversity of microbial samples under both 16S rRNA gene sequencing and metagenomic sequencing. Fig. S5. The beta diversity of microbial samples under both 16S rRNA gene sequencing and metagenomic sequencing. Fig. S6. Microbial composition at the phylym level that calculated by metagenomic sequenceing data. Fig. S7. The abundance of species that were specific biomarkers taxa at each time points. Fig. S8. Richness of functional gene in microbial samples at three time points. Fig. S9. Distribution of Ruminococcus in microbial samples at three time points. [file 40104_2023_943_MOESM2_ESM.zip › Figure S3.jpg]

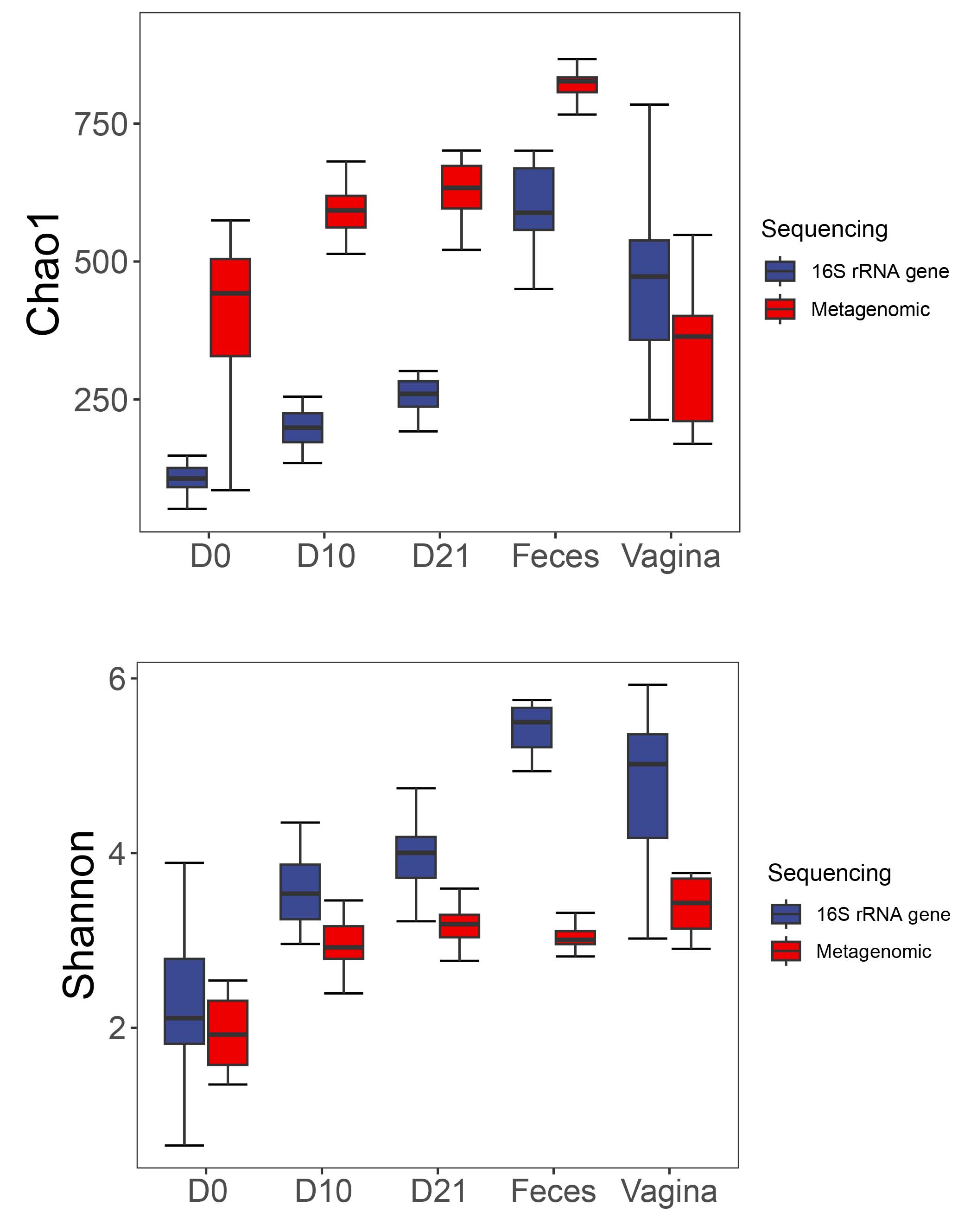

Supplement: Supplementary file 2 — Additional file 2: Fig. S1. Alpha diversity of microbial community among different groups based on metagenomic sequencing data. Fig. S2. NMDS plot according sample group based on the abundance of species. Fig. S3. The mean of ranked dissimilarities between groups to the mean of ranked dissimilarities within groups. Fig. S4. The alpha diversity of microbial samples under both 16S rRNA gene sequencing and metagenomic sequencing. Fig. S5. The beta diversity of microbial samples under both 16S rRNA gene sequencing and metagenomic sequencing. Fig. S6. Microbial composition at the phylym level that calculated by metagenomic sequenceing data. Fig. S7. The abundance of species that were specific biomarkers taxa at each time points. Fig. S8. Richness of functional gene in microbial samples at three time points. Fig. S9. Distribution of Ruminococcus in microbial samples at three time points. [file 40104_2023_943_MOESM2_ESM.zip › Figure S4.jpg]

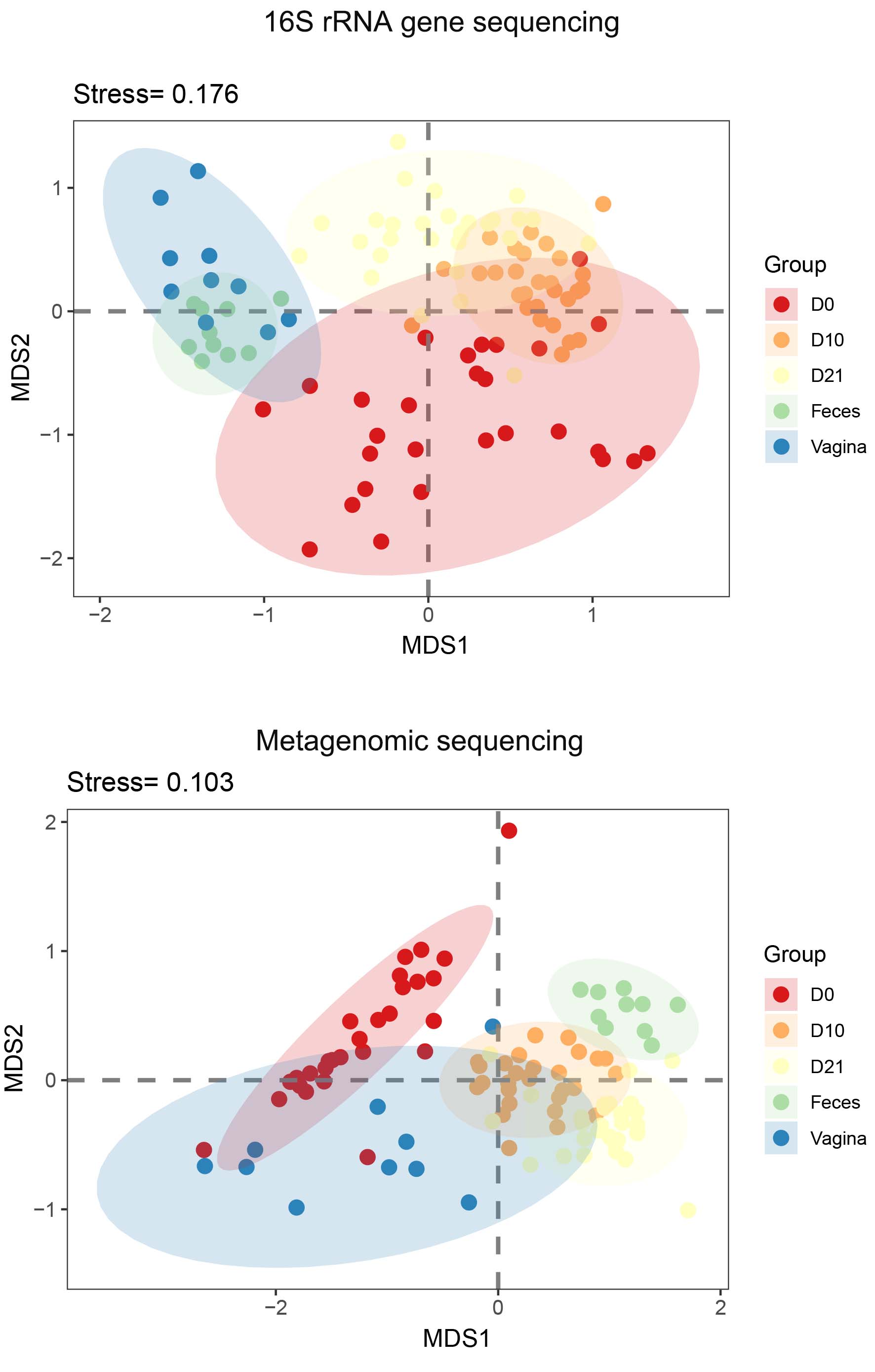

Supplement: Supplementary file 2 — Additional file 2: Fig. S1. Alpha diversity of microbial community among different groups based on metagenomic sequencing data. Fig. S2. NMDS plot according sample group based on the abundance of species. Fig. S3. The mean of ranked dissimilarities between groups to the mean of ranked dissimilarities within groups. Fig. S4. The alpha diversity of microbial samples under both 16S rRNA gene sequencing and metagenomic sequencing. Fig. S5. The beta diversity of microbial samples under both 16S rRNA gene sequencing and metagenomic sequencing. Fig. S6. Microbial composition at the phylym level that calculated by metagenomic sequenceing data. Fig. S7. The abundance of species that were specific biomarkers taxa at each time points. Fig. S8. Richness of functional gene in microbial samples at three time points. Fig. S9. Distribution of Ruminococcus in microbial samples at three time points. [file 40104_2023_943_MOESM2_ESM.zip › Figure S5.jpg]

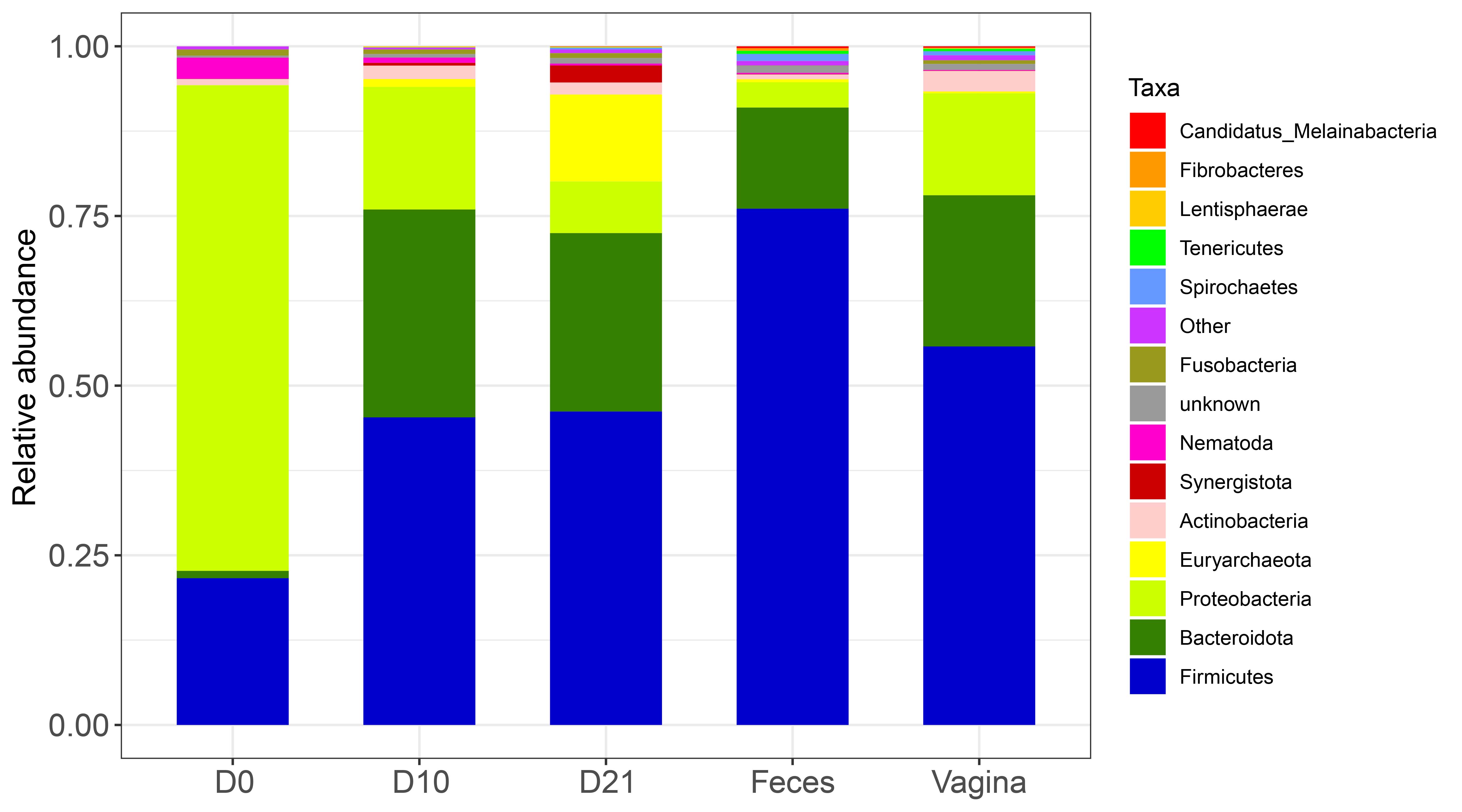

Supplement: Supplementary file 2 — Additional file 2: Fig. S1. Alpha diversity of microbial community among different groups based on metagenomic sequencing data. Fig. S2. NMDS plot according sample group based on the abundance of species. Fig. S3. The mean of ranked dissimilarities between groups to the mean of ranked dissimilarities within groups. Fig. S4. The alpha diversity of microbial samples under both 16S rRNA gene sequencing and metagenomic sequencing. Fig. S5. The beta diversity of microbial samples under both 16S rRNA gene sequencing and metagenomic sequencing. Fig. S6. Microbial composition at the phylym level that calculated by metagenomic sequenceing data. Fig. S7. The abundance of species that were specific biomarkers taxa at each time points. Fig. S8. Richness of functional gene in microbial samples at three time points. Fig. S9. Distribution of Ruminococcus in microbial samples at three time points. [file 40104_2023_943_MOESM2_ESM.zip › Figure S6.jpg]

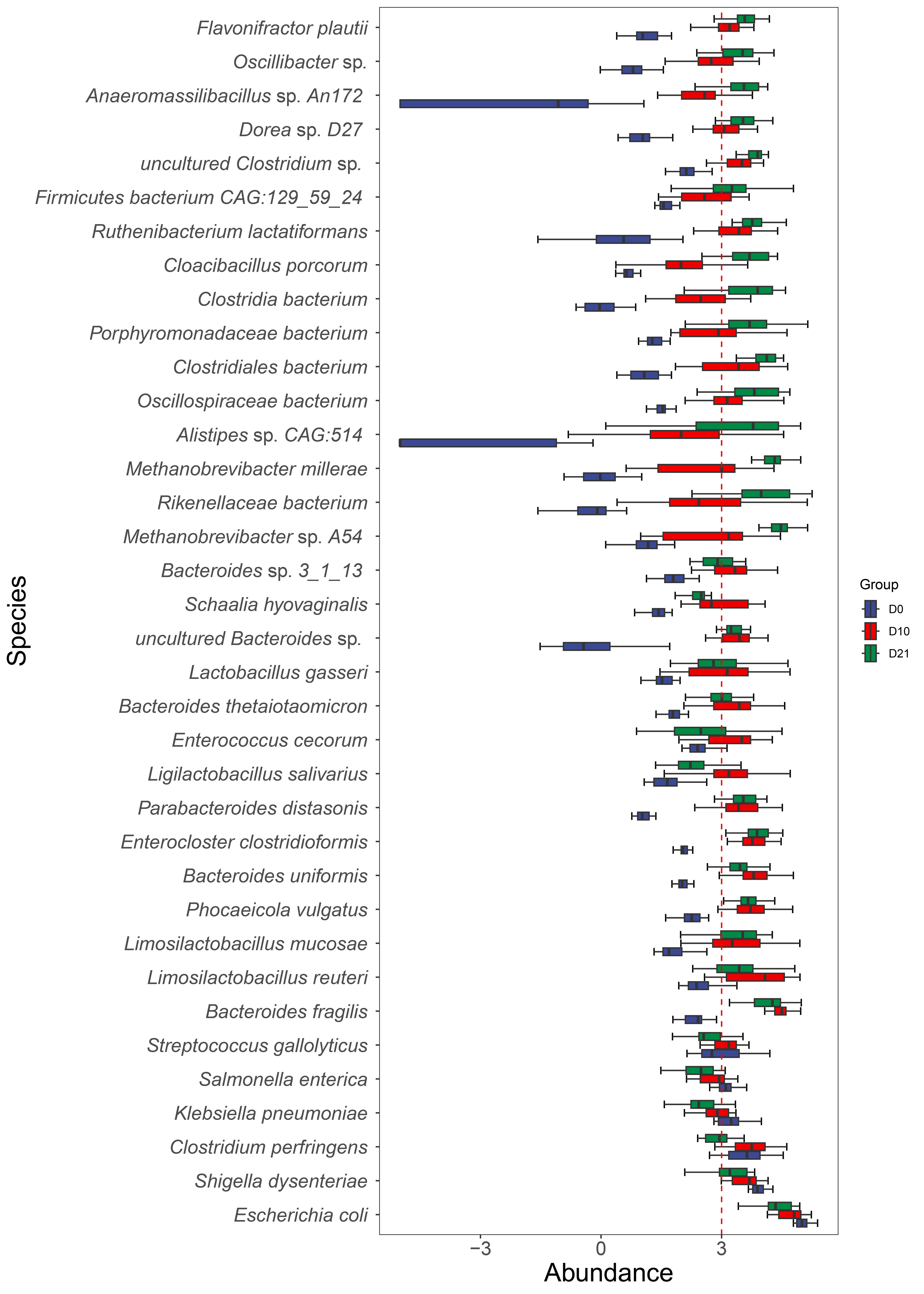

Supplement: Supplementary file 2 — Additional file 2: Fig. S1. Alpha diversity of microbial community among different groups based on metagenomic sequencing data. Fig. S2. NMDS plot according sample group based on the abundance of species. Fig. S3. The mean of ranked dissimilarities between groups to the mean of ranked dissimilarities within groups. Fig. S4. The alpha diversity of microbial samples under both 16S rRNA gene sequencing and metagenomic sequencing. Fig. S5. The beta diversity of microbial samples under both 16S rRNA gene sequencing and metagenomic sequencing. Fig. S6. Microbial composition at the phylym level that calculated by metagenomic sequenceing data. Fig. S7. The abundance of species that were specific biomarkers taxa at each time points. Fig. S8. Richness of functional gene in microbial samples at three time points. Fig. S9. Distribution of Ruminococcus in microbial samples at three time points. [file 40104_2023_943_MOESM2_ESM.zip › Figure S7.jpg]

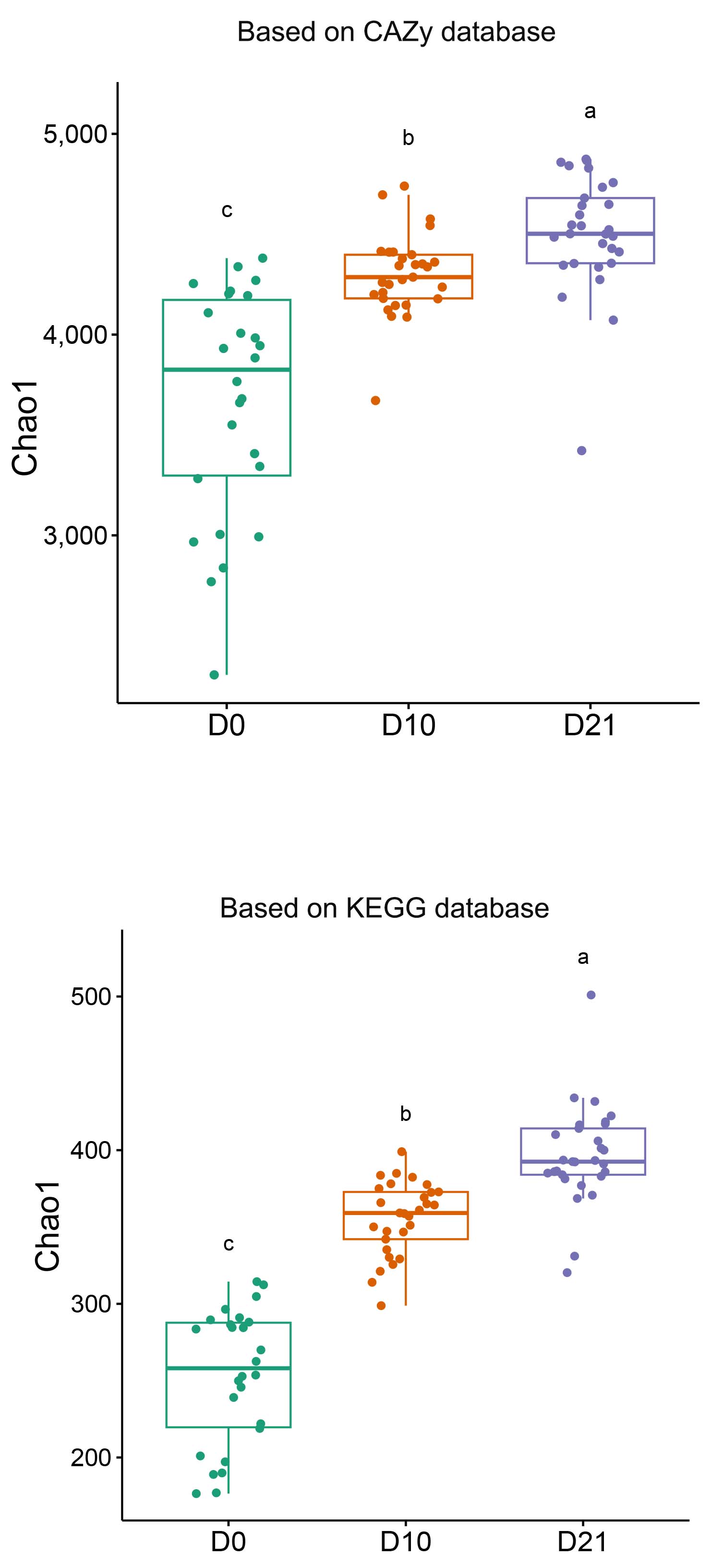

Supplement: Supplementary file 2 — Additional file 2: Fig. S1. Alpha diversity of microbial community among different groups based on metagenomic sequencing data. Fig. S2. NMDS plot according sample group based on the abundance of species. Fig. S3. The mean of ranked dissimilarities between groups to the mean of ranked dissimilarities within groups. Fig. S4. The alpha diversity of microbial samples under both 16S rRNA gene sequencing and metagenomic sequencing. Fig. S5. The beta diversity of microbial samples under both 16S rRNA gene sequencing and metagenomic sequencing. Fig. S6. Microbial composition at the phylym level that calculated by metagenomic sequenceing data. Fig. S7. The abundance of species that were specific biomarkers taxa at each time points. Fig. S8. Richness of functional gene in microbial samples at three time points. Fig. S9. Distribution of Ruminococcus in microbial samples at three time points. [file 40104_2023_943_MOESM2_ESM.zip › Figure S8.jpg]

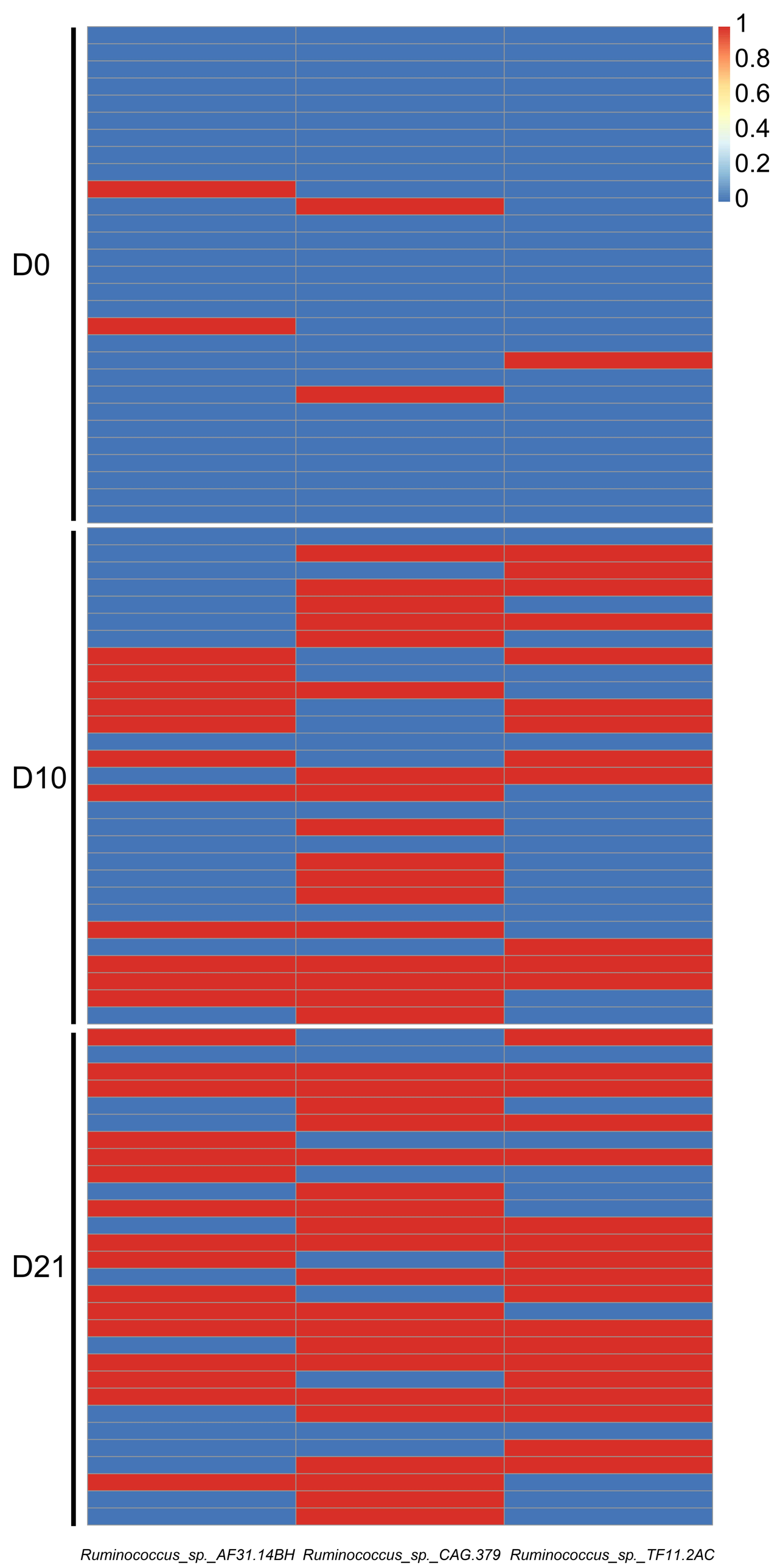

Supplement: Supplementary file 2 — Additional file 2: Fig. S1. Alpha diversity of microbial community among different groups based on metagenomic sequencing data. Fig. S2. NMDS plot according sample group based on the abundance of species. Fig. S3. The mean of ranked dissimilarities between groups to the mean of ranked dissimilarities within groups. Fig. S4. The alpha diversity of microbial samples under both 16S rRNA gene sequencing and metagenomic sequencing. Fig. S5. The beta diversity of microbial samples under both 16S rRNA gene sequencing and metagenomic sequencing. Fig. S6. Microbial composition at the phylym level that calculated by metagenomic sequenceing data. Fig. S7. The abundance of species that were specific biomarkers taxa at each time points. Fig. S8. Richness of functional gene in microbial samples at three time points. Fig. S9. Distribution of Ruminococcus in microbial samples at three time points. [file 40104_2023_943_MOESM2_ESM.zip › Figure S9.jpg]
